# Identification and Classification of Hubs in microRNA Target Gene Networks in Human Neural Stem/Progenitor Cells following Japanese Encephalitis Virus Infection

**DOI:** 10.1128/mSphere.00588-19

**Published:** 2019-10-02

**Authors:** Sriparna Mukherjee, Irshad Akbar, Reshma Bhagat, Bibhabasu Hazra, Arindam Bhattacharyya, Pankaj Seth, Dipanjan Roy, Anirban Basu

**Affiliations:** a National Brain Research Centre, Manesar, Haryana, India; b Immunology Lab, Department of Zoology, University of Calcutta, Kolkata, India; Wake Forest University

**Keywords:** microRNA, Japanese encephalitis virus, PPI network, hub genes, miRNA

## Abstract

RNA viruses are known to modulate host microRNA (miRNA) machinery for their own benefit. Japanese encephalitis virus (JEV), a neurotropic RNA virus, has been reported to manipulate several miRNAs in neurons or microglia. However, no report indicates a complete sketch of the miRNA profile of neural stem/progenitor cells (NSPCs), hence the focus of our current study. We used an miRNA array of 84 miRNAs in uninfected and JEV-infected human neuronal progenitor cells and primary neural precursor cells isolated from aborted fetuses. Severalfold downregulation of hsa-miR-9-5p, hsa-miR-22-3p, hsa-miR-124-3p, and hsa-miR-132-3p was found postinfection in both of the cell types compared to the uninfected cells. Subsequently, we screened for the target genes of these miRNAs and looked for the biological pathways that were significantly regulated by the genes. The target genes involved in two or more pathways were sorted out. Protein-protein interaction (PPI) networks of the miRNA target genes were formed based on their interaction patterns. A binary adjacency matrix for each gene network was prepared. Different modules or communities were identified in those networks by community detection algorithms. Mathematically, we identified the hub genes by analyzing their degree centrality and participation coefficient in the network. The hub genes were classified as either provincial (*P* < 0.4) or connector (*P* > 0.4) hubs. We validated the expression of hub genes in both cell line and primary cells through qRT-PCR after JEV infection and respective miR mimic transfection. Taken together, our findings highlight the importance of specific target gene networks of miRNAs affected by JEV infection in NSPCs.

**IMPORTANCE** JEV damages the neural stem/progenitor cell population of the mammalian brain. However, JEV-induced alteration in the miRNA expression pattern of the cell population remains an open question, hence warranting our present study. In this study, we specifically address the downregulation of four miRNAs, and we prepared a protein-protein interaction network of miRNA target genes. We identified two types of hub genes in the PPI network, namely, connector hubs and provincial hubs. These two types of miRNA target hub genes critically influence the participation strength in the networks and thereby significantly impact up- and downregulation in several key biological pathways. Computational analysis of the PPI networks identifies key protein interactions and hubs in those modules, which opens up the possibility of precise identification and classification of host factors for viral infection in NSPCs.

## Abstract (precis)

JEV damages the neural stem/progenitor cell population of the mammalian brain. However, JEV-induced alteration in the miRNA expression pattern of the cell population remains an open question, hence warranting our present study. In this study, we specifically address the downregulation of four miRNAs, and we prepared a protein-protein interaction network of miRNA target genes. We identified two types of hub genes in the PPI network, namely, connector hubs and provincial hubs. These two types of miRNA target hub genes critically influence the participation strength in the networks and thereby significantly impact up- and downregulation in several key biological pathways. Computational analysis of the PPI networks identifies key protein interactions and hubs in those modules, which opens up the possibility of precise identification and classification of host factors for viral infection in NSPCs.

## INTRODUCTION

Japanese encephalitis virus (JEV), transmitted by an arthropod vector, effectively replicates inside the central nervous system (CNS) and causes neuronal death. JEV also infects the mitotically active neural stem/progenitor cell (NSPC) population residing in the subventricular zone (SVZ) and creates imbalance in endoplasmic reticulum homeostasis, thus activating cell death pathways (1, 2). Cellular damage in the CNS is reversed by migration of new cells to the injury site from the neurogenic niche (3, 4). Thus, JEV promotes a double injury to the brain by damaging the neurons and diminishing the stem cell pool as well, so that the CNS repair mechanism is hindered. JEV infection leads to severe cognitive and motor deficiencies in survivors. Hence, NSPC depletion is thought to be the most important contributing factor responsible for neurological sequelae observed in JE survivors. On that premise, microRNAs (miRNAs) play a pivotal role in CNS development. Several miRNAs have been reported to regulate neurogenesis, neuronal migration, gliogenesis, synaptogenesis, long-term potentiation, and synaptic plasticity (5). They also regulate neural stem cell self-renewal and fate determination (6), the processes evidently affected by JEV infection. Therefore, investigation of the NSPC miRNA profile upon neurotropic virus invasion is of utmost importance.

The first miRNA was discovered in 1993 (7). Since then, radical research progress has been made in discovering their synthesis and biological importance. These 20- to 25-nucleotide (nt)-long RNAs have been shown to bind to the 3′ untranslated region (UTR) of target genes and thus restrain their translation (8). The small noncoding RNAs fine-tune critical cellular processes in stem cells, including cell cycle control, proliferation, differentiation, and apoptosis (9). Aberrant expression of various miRNAs is documented in cases of Alzheimer’s disease (10), Parkinson’s disease (11), amyotrophic lateral sclerosis (12), schizophrenia (13), and autism (14). miRNAs function as disease biomarkers, and monitoring miRNA levels in a biological system provides a comprehensive picture of disease progression. Since miRNAs govern the expression of 50% of protein coding genes in mammals, targeting them in a disease state is an appealing therapeutic strategy (15).

Tissue-specific miRNA response upon viral challenge can directly alter viral replication and pathogenesis. Several studies report a close connection of RNA virus infection and modulation of host miRNAs (16, 17). For example, infection by Eastern equine encephalitis virus (an alphavirus) modulates miR-142-3p expression in human and mouse macrophages (18), thus affecting viral propagation. Upon picornavirus and orthomyxovirus infections, host miRNA modifications have been observed (19, 20). Neurotropic viruses can exploit the miRNA machinery in NSPCs. Downregulation of miR-155 has been observed in HIV infection in neural precursor cells (21). miR-15b (22), miR-29b, and miR-155 were found to regulate JEV-induced neuroinflammation (23, 24). A recent study also indicates a role for miR-301a in inhibiting type I interferon signaling during JEV infection, thus regulating host immune response (25). However, a clear understanding regarding miRNA response in NSPCs after JEV infection is still lacking.

Our group employed an *in silico* tool to assess miRNA alteration following JEV infection in both hNS1 cells and neural precursor cells isolated from aborted human fetuses. Four miRNAs, namely, hsa-miR-9-5p, hsa-miR-22-3p, hsa-miR-124-3p, and hsa-miR-132-3p, were found to be consistently downregulated in both the cell types upon JEV infection, hence being the focus of our study. Through an analysis including target prediction, community detection, miRNA target hub gene identification, and molecular validation, we illustrated miRNA-target gene interaction in cases of JEV infection. These miRNAs might promote viral propagation and persistence in NSPCs, thus warranting further studies. We also anticipate that our pilot study will open up the possibility of precise identification and classification of primary interaction partners of miRNAs playing roles as the key host factors for viral infection in NSPCs.

(This article was submitted to an online preprint archive [26].)

## RESULTS

### JEV infection alters miRNA expression profile of NSPCs.

**FIG 1:**
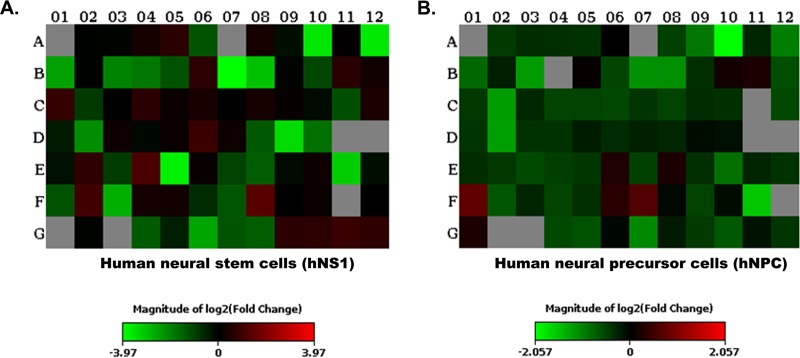
Heat map depicting differential expression of miRNAs in hNS1 and hNP cells. miRNA was extracted from mock-infected (control) and JEV-infected hNS1 and hNPC samples and analyzed with the human neurological development and disease miRNA PCR array. (A) Heat map showing differential expression of miRNAs in JEV-infected hNS1 cells compared to mock-infected cells. (B) Heat map showing differential expression of miRNAs in JEV-infected hNP cells compared to control. Analysis was performed using PCR array data analysis from SABiosciences. Data are representative of three independent experiments. Colors indicate the fold change value (green, downregulation; red, upregulation).

miRNAs isolated from mock-infected (control) and JEV-infected hNS1 and hNPC samples were analyzed with the human neurological development and disease miRNA PCR array (Qiagen product no. 331221, catalog number MIHS-107Z) in order to find the miRNAs differentially expressed upon viral infection. The majority of the miRNAs were found to be downregulated in both the cell types upon infection (Fig. 1). Colored squares in Fig. 1 are indicative of fold change values for each of the miRNA in the infected cells compared to mock-infected cells. The statistical values of the raw data obtained from the array plates were calculated using Qiagen data analysis software available online, and the analysis was done by the system itself, after which Tables S2 and S3 in the supplemental material were generated. Tables S2 and S3 show the name and fold change value of the 84 miRNAs present in the mentioned array. Among the downregulated miRNAs, we sorted out the ones that were downregulated in both the cell types, and thus, hsa-miR-9-5p, hsa-miR-22-3p, hsa-miR-124-3p, and hsa-miR-132-3p were chosen for further experiments.

### Analysis of target gene networks and hub identification for all four miRNAs.

**FIG 2:**
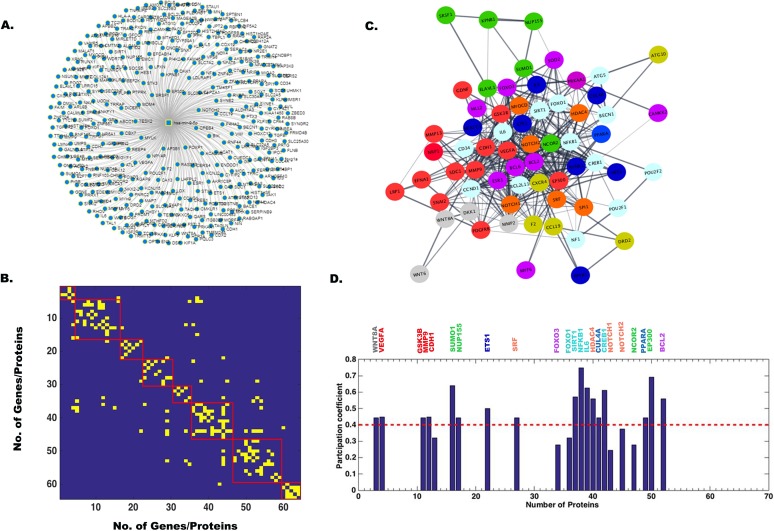
Network analysis showing miR-9-5p target genes, modularity/community detection in gene networks, and the identification of hub genes based on participation coefficients. (A) A comprehensive human miRNA-gene network constructed by miRnet.ca database. In String database, 60 genes were found to be connected, and based on their interaction, an adjacency matrix *A_ij_* was formed, which was used for network analysis. This adjacency matrix *A_ij_* comprises 60 target genes (nodes) and binarized edges based on their functional annotations (B). Modularity score *Q* was computed using the Brain Connectivity Toolbox (BCT) in Matlab, and genes were partitioned into eight identified communities based on similarity of connection patterns for each pair of vertices/nodes (C). Based on the node degree *kv* of node *v*, and *kvs* being the number of edges of the *v*th node to nodes within module *s*, we have estimated each vertex’s participation index *P* and quantified the presence of provincial and connector hub genes (D). The participation index *P* shows that there are provincial and connector hub genes belonging to eight communities.

**FIG 3:**
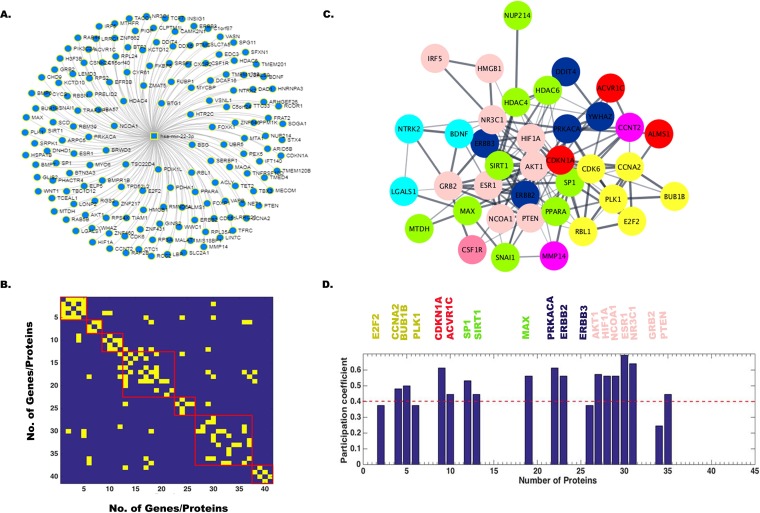
Network analysis showing miR-22-3p target genes, modularity/community detection in gene networks, and the identification of hub genes based on participation coefficients. (A) A comprehensive human miRNA-gene network constructed in miRnet.ca database. In String database, 40 genes were found to be connected, and based on their interaction, an adjacency matrix *A_ij_* was formed, which was used for network analysis. This adjacency matrix *A_ij_* comprises 40 target genes (nodes) and binarized edges based on their functional annotations (B). Modularity score *Q* was computed using the Brain Connectivity Toolbox (BCT) in Matlab, and genes were partitioned into seven identified communities based on similarity of connection patterns for each pair of vertices/nodes (C). Based on the node degree *kv* of node *v*, and *kvs* being the number of edges from the *v*th node to nodes within module *s*, we have estimated each vertex’s participation index *P* and quantified the presence of provincial and connector hub genes (D). The participation index *P* shows that there are provincial and connector hub genes belonging to seven communities.

**FIG 4:**
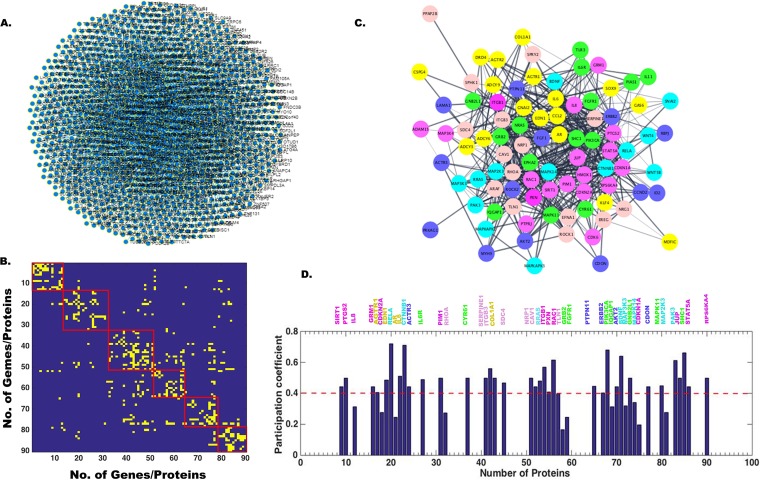
Network analysis showing miR-124-3p target genes, modularity/community detection in gene networks, and the identification of hub genes based on participation coefficients. (A) A comprehensive human miRNA-gene network constructed in miRnet.ca database. In String database, 90 genes were found to be connected, and based on their interaction, an adjacency matrix *A_ij_* was formed, which was used for network analysis. This adjacency matrix *A_ij_* comprises 90 target genes (nodes) and binarized edges based on their functional annotations (B). Modularity score *Q* was computed using the Brain Connectivity Toolbox (BCT) in Matlab, and genes were partitioned into six identified communities based on similarity of connection patterns for each pair of vertices/nodes (C). Based on the node degree *kv* of node *v*, and *kvs* being the number of edges from the *v*th node to nodes within module *s*, we have estimated each vertex’s participation index *P* and quantified the presence of provincial and connector hub genes (D). The participation index *P* shows that there are provincial and connector hub genes belonging to all the identified six communities.

**FIG 5:**
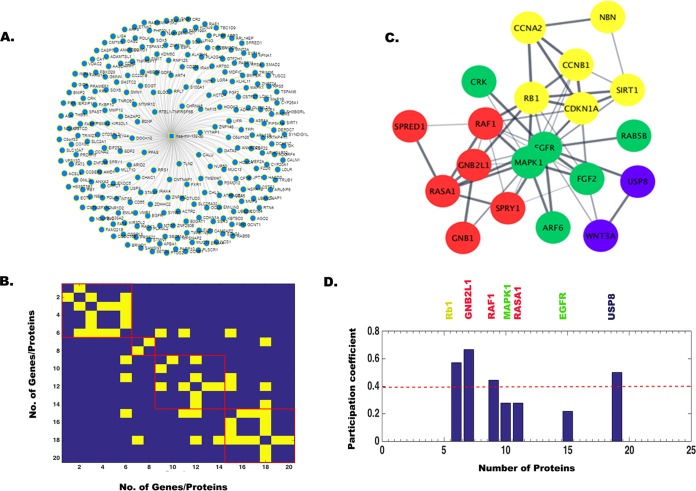
Network analysis showing miR-132-3p target genes, modularity/community detection in gene networks, and the identification of hub genes based on participation coefficients. (A) A comprehensive human miRNA-gene network constructed in miRnet.ca database. In String database, 20 genes were found to be connected, and based on their interaction, an adjacency matrix *A_ij_* was formed, which was used for network analysis. This adjacency matrix *A_ij_* comprises 20 target genes (nodes) and binarized edges based on their functional annotations (B). Modularity score *Q* was computed using the Brain Connectivity Toolbox (BCT) in Matlab, and genes were partitioned into four identified communities based on similarity of connection patterns for each pair of vertices/nodes (C). Based on the node degree *kv* of node *v*, and *kvs* being the number of edges from the *v*th node to nodes within module *s*, we have estimated each vertex’s participation index *P* and quantified the presence of provincial and connector hub genes (D). The participation index *P* shows that there are provincial and connector hub genes belonging to all the identified four communities.

In order to identify target genes of all four miRNAs, hsa-miR-9-5p, hsa-miR-22-3p, hsa-miR-124-3p, and hsa-miR-132-3p, we used miRnet.ca and String 9.1 online database (http://string-db.org/) for extraction of the gene networks. These gene names and their repetition number in various pathways are listed in Table S4. There were about 60 target genes for hsa-miR-9-5p, 40 target genes for hsa-miR-22-3p, 90 target genes for hsa-miR-124-3p, and 20 target genes for hsa-miR-132-3p identified using primarily keyword-based searching from a list using the String database. Next, a comprehensive human gene-gene network was constructed for all four miRNAs by defining an adjacency matrix *A_ij_* which was subsequently used for the network analysis. This adjacency matrix *A_ij_* comprises 60, 40, 90, and 20 target genes (nodes) for each of the four miRNAs, respectively. The binarized edges based on their functional annotations are displayed in Fig. 2A and B for hsa-miR-9-5p and similarly for other miRNAs in Fig. 3A and B, Fig. 4A and B, and Fig. 5A and B. Subsequently, modularity score *Q* was computed using the Brain Connectivity Toolbox (BCT) in Matlab, and genes were partitioned into eight identified communities based on similarity of connection patterns for each pair of vertices/nodes based on equations 1, 2, and 3 and plotted in Fig. 2C. Similarly, hsa-miR-22-3p target genes were partitioned into seven identified communities as shown in Fig. 3C. Repeating the above analysis on hsa-miR-124-3p and hsa-miR-132-3p, we found six and four communities, respectively, as shown in Fig. 4C and Fig. 5C, respectively. This process of community detection was repeated using the greedy search procedure based on the Louvain method using equations 4, 5, and 6. In order to visualize these communities identified for all four miRNAs and their interactomes, Cytoscape (https://cytoscape.org/), an open-source online platform, was used to study community-level specific interaction patterns. The network modularity analysis further confirmed that there were several dominant players in the biological pathway underlying JEV infection in neurons for all four miRNAs. Furthermore, in order to find out which genes may serve as hub genes and modulate more than one subnetwork, we have employed a hub identification algorithm. Based on the node degree *k_v_* of node *v*, and *k_vs_* being the number of edges from the *v*th node to nodes within module *s*, we have estimated each vertex’s participation index *P* and quantified the presence of provincial and connector hub genes in the interactome network. The results of hub analysis for all four miRNAs are displayed in Fig. 2D, Fig. 3D, Fig. 4D, and Fig. 5D, respectively. Our hub analysis predicted that there were a number of connector hub genes present in each module, including SIRT1, PTGS2, ETS1, SUMO1, and IL-6 for hsa-miR-9-5p. Similarly, this analysis revealed CDKN1A, SIRT1, NR3C1, MAX1, NCOA1, ESR1, SP1, PTEN, etc., being specifically targeted by the hsa-miR-22-3p. We also found, based on hub classification, connector hub genes, e.g., SDC4, PIK3CA, CAV1, PIM1, IQGAP1, NRP1, SHC1, MAP3K3, etc., specifically targeted by the hsa-miR-124-3p, and, similarly, a number of connector hub genes such as RAF1, Rb1, MAPK1, RASA1, EGFR1, USP8, etc., specifically targeted by the hsa-miR-132-3p. Next, we carried out a molecular validation assay to see whether any of the identified hub genes became up- or downregulated after JEV infection in different cell types.

### Expression of miR target genes in hNS1 and hNP cells upon JEV infection and mimic transfection.

The hub genes of the four miRNAs were analyzed in TargetScan (http://www.targetscan.org) in order to identify any binding site of the miRNA in the 3′ UTR of target genes before validation through qRT-PCR and mimic treatment in both hNS1 and hNP cells. TargetScan is a well-known software program which predicts effective target sites of miRNAs on mammalian mRNA. Interaction of miR-9, -22, and -124 with certain hub genes was found in TargetScan and hence validated. However, no interaction of miR-132-3p and its hub genes was identified in the database, and hence no validation study was done. Optimization of miR mimic treatment was done in both the cell types prior to JEV infection, and qRT-PCR was performed to assess the upregulation of miRNAs upon mimic treatment (see Fig. S1 in the supplemental material).

10.1128/mSphere.00588-19.1 FIG S1 Optimization of miR-mimic treatment in both hNS1 and hNP cells. Both hNS1 and hNP cells were transfected with 30 nM mimic of hsa-miR-9-5p, hsa-miR-22-3p, hsa-miR-124-3p, and hsa-miR-132-3p using HiPerFect transfection reagent. At 24 h posttransfection, cells were harvested for miRNA isolation and validation of transfection through qRT-PCR using primers of respective miRNAs. The upper panel shows significant upregulation of 4 miRNAs in hNS1 cells compared to mock infection, and the lower panel indicates the same in hNP cells. M, mock transfection; MC, control (nonspecific) mimic transfection. Data are representative of three independent experiments (mean ± SD). *, *P* < 0.05; **, *P* < 0.01. *P* value is calculated by one-way ANOVA followed by Bonferroni *post hoc* test. Download FIG S1, TIF file, 0.6 MB. Copyright © 2019 Mukherjee et al. 2019 Mukherjee et al. This content is distributed under the terms of the Creative Commons Attribution 4.0 International license.

**FIG 6:**
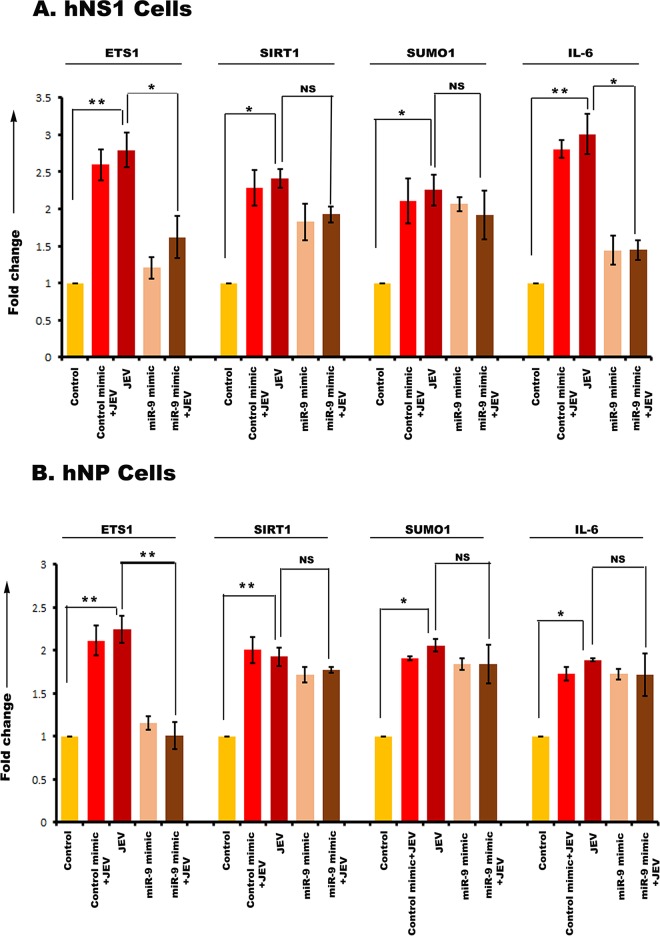
qRT-PCR showing miR-9-5p target gene expression in hNS1 and hNP cells upon JEV infection and miR mimic transfection. RNA isolated from control, control mimic-transfected plus JEV-infected, JEV-infected, miR-9-5p mimic-transfected, and miR-9-5p mimic-transfected plus JEV-infected hNS1and hNP cells was subjected to qRT-PCR. JEV infection led to significant upregulation of ETS1, SIRT1, SUMO1, and IL-6 in both the cell types compared to control (A and B). However, upon mimic transfection and JEV infection, ETS1 and IL-6 genes showed a reduced expression profile in hNS1 cells (A), whereas, in the case of hNP cells, only ETS1 gene expression was downregulated after mimic transfection and viral infection (B). In both cases, fold change in gene expression was assessed between JEV and JEV plus miR mimic groups. GAPDH gene expression was used as an internal control for normalization of PCR data in both the cell types. Data are representative of three independent experiments (mean ± SD) by one-way ANOVA (*, *P* < 0.05; **, *P* < 0.01; NS, not significant).

**FIG 7:**
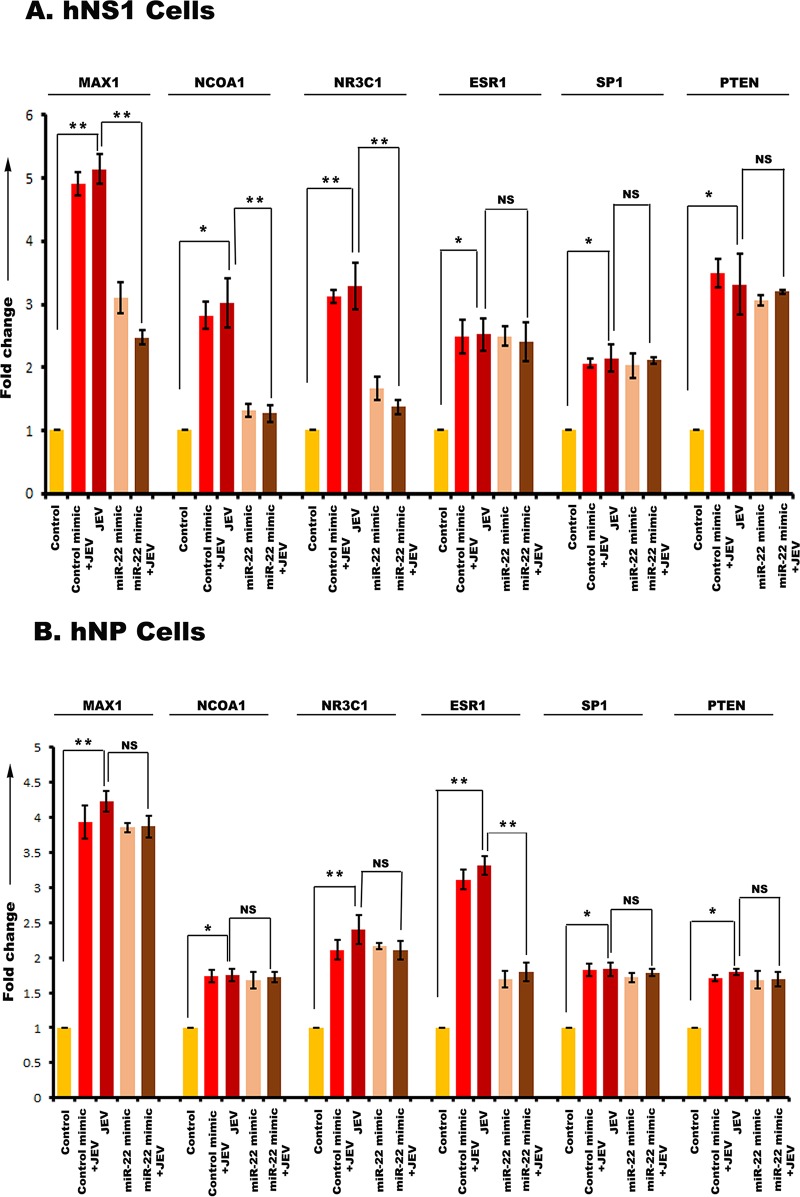
qRT-PCR showing miR-22-3p target gene expression in hNS1 and hNP cells upon JEV infection and miR mimic transfection. RNA isolated from control, control mimic-transfected plus JEV-infected, JEV-infected, miR-22-3p mimic-transfected, and miR-22-3p mimic-transfected plus JEV-infected hNS1 and hNP cells was subjected to qRT-PCR. JEV infection led to significant upregulation of MAX1, NCOA1, NR3C1, ESR1, SP1, and PTEN in both the cell types compared to control (A and B). However, upon mimic transfection and JEV infection, MAX1, NCOA1, and NR3C1 genes showed a reduced expression profile in hNS1 cells (A), whereas in the case of hNP cells, only ESR1 gene expression was downregulated after mimic transfection and viral infection (B). In both cases, fold change in gene expression was assessed between JEV and JEV plus miR mimic groups. GAPDH gene expression was used as an internal control for normalization of PCR data in both the cell types. Data are representative of three independent experiments (mean± SD) by one-way ANOVA (*, *P* < 0.05; **, *P* < 0.01; NS, not significant).

**FIG 8:**
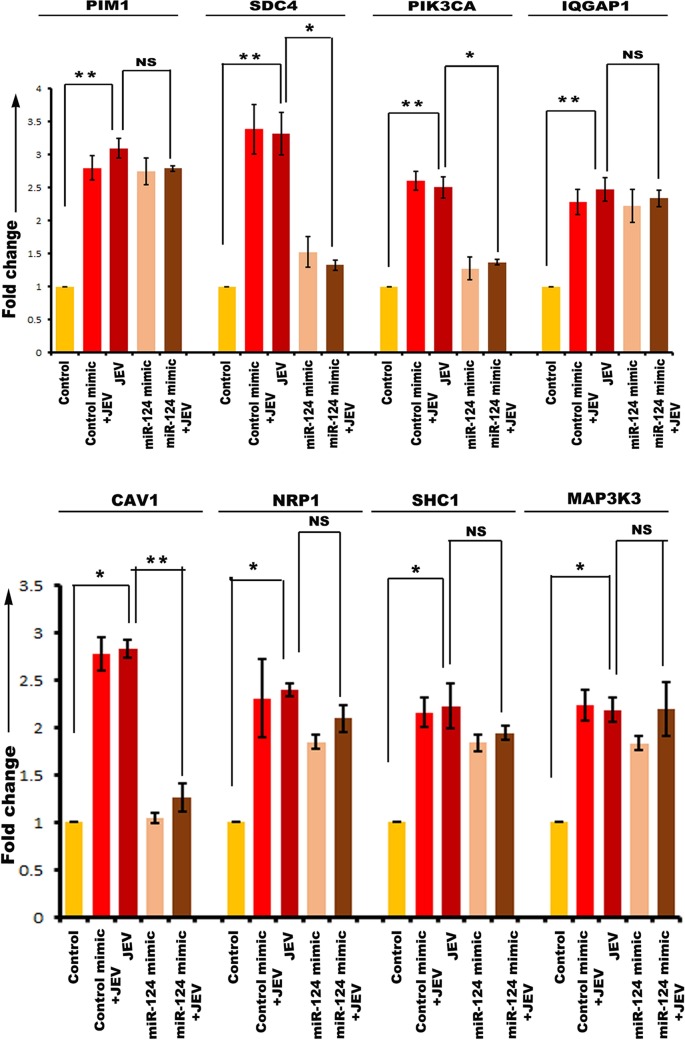
qRT-PCR showing miR-124-3p target gene expression in hNS1 cells upon JEV infection and miR mimic transfection. RNA isolated from control, control mimic-transfected plus JEV-infected, JEV-infected, miR-124-3p mimic-transfected, and miR-124-3p mimic-transfected plus JEV-infected hNS1 cells was subjected to qRT-PCR. JEV infection led to significant upregulation of PIM1, SDC4, PIK3CA, and IQGAP1 (upper panel) and CAV1, NRP1, SHC1, and MAP3K3 (lower panel) compared to control. However, upon mimic transfection and JEV infection, SDC4, PIK3CA, and CAV1 genes showed a reduced expression profile compared to that between JEV and JEV plus miR mimic groups. GAPDH gene expression was used as an internal control for normalization of PCR data. Data are representative of three independent experiments (mean ± SD) by one-way ANOVA (*, *P* < 0.05; **, *P* < 0.01; NS, not significant).

**FIG 9:**
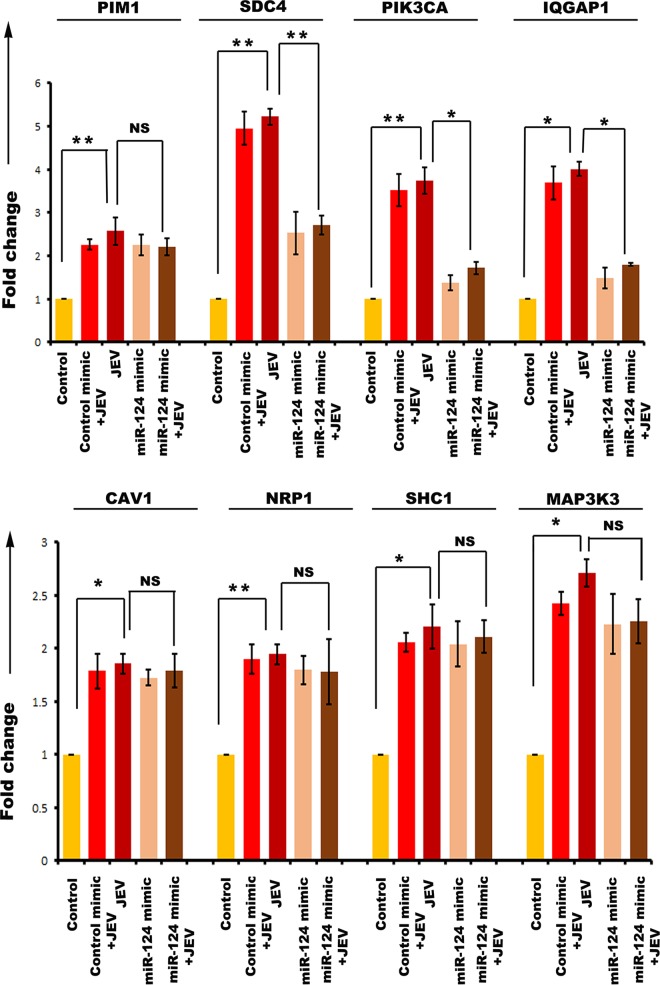
qRT-PCR showing miR-124-3p target gene expression in hNP cells upon JEV infection and miR mimic transfection. RNA isolated from control, control mimic-transfected plus JEV-infected, JEV-infected, miR-124-3p mimic-transfected, and miR-124-3p mimic-transfected plus JEV-infected hNP cells was subjected to qRT-PCR. JEV infection led to significant upregulation of PIM1, SDC4, PIK3CA, and IQGAP1 (upper panel) and CAV1, NRP1, SHC1, and MAP3K3 (lower panel) compared to control. However, upon mimic transfection and JEV infection, SDC4, PIK3CA, and IQGAP1 genes showed a reduced expression profile compared with that between JEV and JEV plus miR mimic groups. GAPDH gene expression was used as an internal control for normalization of PCR data. Data are representative of three independent experiments (mean ± SD) by one-way ANOVA (*, *P* < 0.05; **, *P* < 0.01; NS, not significant).

Then, hNS1 and hNP cells were divided into 5 experimental groups. One group was transfected with control mimic, and two other groups were transfected with the respective miR mimics (miR-9-5p mimic, miR-22-3p mimic, miR-123-3p mimic, and miR-132-3p mimic). At 24 h posttransfection, 3 groups were infected at a multiplicity of infection (MOI) of 5 with JEV (JEV, control mimic plus JEV, and miR mimic plus JEV). The control group was treated with an equal volume of phosphate-buffered saline (PBS). At 72 h postinfection, cells were harvested for RNA isolation and cDNA preparation. Expression of miR-9-5p target genes was validated through qRT-PCR with both hNS1 (Fig. 6A) and hNP (Fig. 6B) cell samples. Although ETS1, SIRT1, SUMO1, and IL-6 had upregulation after JEV infection, miR-9-5p mimic transfection could not downregulate all of their expressions. ETS1 and IL-6 were found to be significantly downregulated upon mimic transfection in hNS1 cells, whereas only ETS1 was notably downregulated in hNP cells under the same experimental condition. Expression of miR-22-3p target genes was validated through qRT-PCR with both hNS1 (Fig. 7A) and hNP (Fig. 7B) cell samples. The target genes MAX1, NCOA1, NR3C1, ESR1, SP1, and PTEN had an upregulation after JEV infection. Upon miR-22-3p mimic transfection, MAX1, NCOA1, and NR3C1 were found to be significantly downregulated in hNS1 cells, whereas only ESR1 was notably downregulated in hNP cells. Similarly, SDC4, PIK3CA, and CAV1 genes showed upregulated expression upon viral infection, and after miR-124-3p mimic transfection and infection, their expression levels were decreased in hNS1 cells (Fig. 8). Other genes (PIM1, IQGAP1, NRP1, SHC1, and MAP3K3) had an upregulated profile postinfection, but miR-124-3p mimic transfection had no effect on their expression. On the other hand, SDC4, PIK3CA, and IQGAP1 genes showed upregulated expression upon viral infection, and after mimic transfection and infection, their expression levels were decreased in hNP cells (Fig. 9). Other genes (PIM1, CAV1, NRP1, SHC1, and MAP3K3) had an upregulated profile postinfection, but mimic transfection had no effect on their expression. Glyceraldehyde-3-phosphate dehydrogenase (GAPDH) gene expression was taken as normalization control in all qRT-PCR experiments.

### Expression of miR target genes in hNS1 and hNP cells upon JEV infection and inhibitor transfection.

Similar sets of experiments were performed in both the cell types after miR inhibitor treatment. Cells were treated with miR inhibitors, and effective downregulation of respective miRs was validated through qRT-PCR experiments (Fig. S2). Expression of ETS1, SIRT1, SUMO1, and IL-6 was analyzed through qRT-PCR after miR-9-5p inhibitor transfection (Fig. S3) in both hNS1 and hNP cells. Similarly, miR-22-3p and miR-124-3p target gene expression was validated after miR inhibitor transfection (Fig. S4, S5, and S6). GAPDH gene expression was taken as normalization control in all qRT-PCR experiments.

10.1128/mSphere.00588-19.2 FIG S2 Optimization of miR inhibitor treatment in both hNS1 and hNP cells. Both hNS1 and hNP cells were transfected with 50 nM inhibitors of hsa-miR-9-5p, hsa-miR-22-3p, hsa-miR-124-3p, and hsa-miR-132-3p using HiPerFect transfection reagent. At 24 h posttransfection, cells were harvested for miRNA isolation and validation of transfection through qRT-PCR using primers of respective miRNAs. The upper panel shows significant downregulation of 4 miRNAs in hNS1 cells compared to mock infection, and the lower panel indicates the same in hNP cells. Mock, mock transfection; IC, control (nonspecific) inhibitor transfection. Data are representative of three independent experiments (mean ± SD). *, *P* < 0.05; **, *P* < 0.01. *P* value is calculated by one-way ANOVA followed by Bonferroni *post hoc* test. Download FIG S2, TIF file, 0.5 MB. Copyright © 2019 Mukherjee et al. 2019 Mukherjee et al. This content is distributed under the terms of the Creative Commons Attribution 4.0 International license.

10.1128/mSphere.00588-19.3 FIG S3 Effect of miR-9-5p inhibitor treatment on target gene expression in both hNS1 and hNP cells. RNA isolated from control/mock, control inhibitor, and hsa-miR-9-5p inhibitor-transfected hNS1 and hNP cells was subjected to qRT-PCR. miR-9-5p inhibitor treatment led to significant upregulation of ETS1, SIRT1, and IL-6 in both hNS1 and hNP cells compared to both mock and control inhibitor transfection. No change was found in SUMO1 expression in both cell types. Data are representative of three independent experiments (mean ± SD). *, *P* < 0.05; **, *P* < 0.01; NS, not significant. *P* value is calculated by one way ANOVA followed by Bonferroni *post hoc* test. Download FIG S3, TIF file, 0.6 MB. Copyright © 2019 Mukherjee et al. 2019 Mukherjee et al. This content is distributed under the terms of the Creative Commons Attribution 4.0 International license.

10.1128/mSphere.00588-19.4 FIG S4 Effect of miR-22-3p inhibitor treatment on target gene expression in both hNS1 and hNP cells. RNA isolated from control/mock, control inhibitor, and hsa-miR-22-3p inhibitor-transfected hNS1 and hNP cells was subjected to qRT-PCR. miR-22-3p inhibitor treatment led to significant upregulation of MAX1, NCOA1, NR3C1, ESR1, and SP1 in both hNS1 and hNP cells compared to both mock and control inhibitor transfection. No change was found in PTEN expression in both cell types. Data are representative of three independent experiments (mean ± SD). *, *P* < 0.05; **, *P* < 0.01; NS, not significant. *P* value is calculated by one-way ANOVA followed by Bonferroni *post hoc* test. Download FIG S4, TIF file, 0.6 MB. Copyright © 2019 Mukherjee et al. 2019 Mukherjee et al. This content is distributed under the terms of the Creative Commons Attribution 4.0 International license.

10.1128/mSphere.00588-19.5 FIG S5 Effect of miR-124-3p inhibitor treatment on target gene expression in hNS1 cells. RNA isolated from control/mock, control inhibitor, and hsa-miR-124-3p inhibitor-transfected hNS1 cells was subjected to qRT-PCR. miR-124-3p inhibitor treatment led to significant upregulation of PIM1, SDC4, PIK3CA, CAV1, NRP1, SHC1, and MAP3K3 compared to both mock and control inhibitor transfection. No change was found in IQGAP1 expression. Data are representative of three independent experiments (mean ± SD). *, *P* < 0.05; **, *P* < 0.01; NS, not significant. *P* value is calculated by one way ANOVA followed by Bonferroni *post hoc* test. Download FIG S5, TIF file, 0.6 MB. Copyright © 2019 Mukherjee et al. 2019 Mukherjee et al. This content is distributed under the terms of the Creative Commons Attribution 4.0 International license.

10.1128/mSphere.00588-19.6 FIG S6 Effect of miR-124-3p inhibitor treatment on target gene expression in hNP cells. RNA isolated from control/mock, control inhibitor, and hsa-miR-124-3p inhibitor-transfected hNP cells was subjected to qRT-PCR. miR-124-3p inhibitor treatment led to significant upregulation of SDC4, PIK3CA, and CAV1 compared to both mock and control inhibitor transfection. No change was found in the expression of the rest of the genes. Data are representative of three independent experiments (mean ± SD). *, *P* < 0.05; **, *P* < 0.01; NS, not significant. *P* value is calculated by one-way ANOVA followed by Bonferroni *post hoc* test. Download FIG S6, TIF file, 0.6 MB. Copyright © 2019 Mukherjee et al. 2019 Mukherjee et al. This content is distributed under the terms of the Creative Commons Attribution 4.0 International license.

### Altered expression of miRNAs in human autopsy tissue of JEV infection cases.

**FIG 10:**
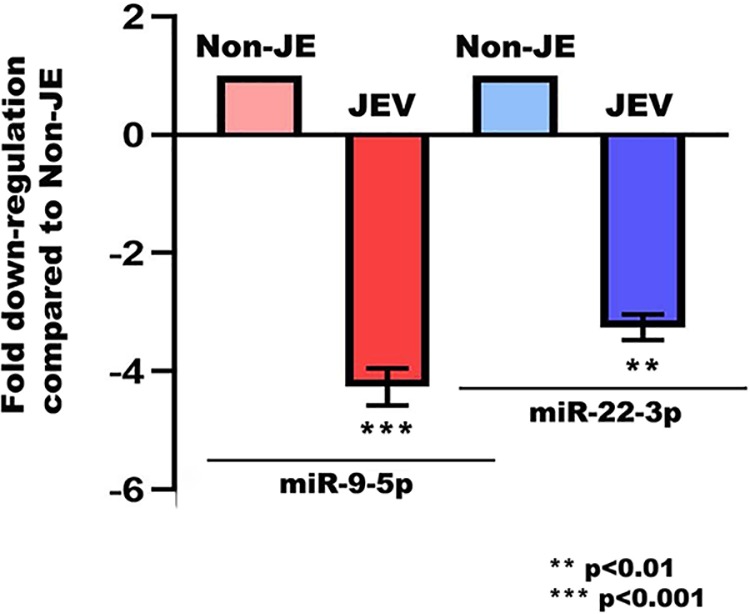
miR-9-5p and miR-22-3p expression in human autopsy tissue from JEV infection. Expression of miR-9-5p and miR-22-3p was analyzed through qRT-PCR in human autopsy tissue from JEV infection and age-matched uninfected (non-JE) tissue. Both of the miRNAs were found to be significantly downregulated in virus-infected cases compared to uninfected tissue. Data are representative of three independent experiments (mean ± SD) by Student’s *t* test (**, *P* < 0.01; ***, *P* < 0.001).

Expression of miR-9-5p, miR-22-3p, miR-124-3p, and miR-132-3p was assessed in JEV-infected and uninfected (non-JE) human autopsy samples. Significant reductions in expression of miR-9-5p and miR-22-3p were found in JEV-infected samples compared to non-JE samples (Fig. 10). Expression levels of other two miRNAs remained unaltered (data not shown). This experiment was performed to obtain parallel data for miRNA profiles in human JE cases. Due to the unavailability of JEV-infected neurogenic region (subventricular zone [SVZ]) in the National Institute of Mental Health and Neurosciences (NIMHANS) brain repository, this experiment was done with basal ganglion tissue. This is an impending limitation to our present study.

## DISCUSSION

miRNAs play a pivotal role in nervous system cell fate determination in worms (27), zebra fish (28), and mammals. miRNAs are found in the mammalian nervous system at higher levels than in the rest of the tissues, the probable reason being its cellular diversity. The same miRNAs can be expressed differently in the different anatomical regions of the brain and again differently during developmental stages. Neurotropic RNA viruses are capable of modulating host miRNA machinery in order to invade and persist in the host system. Changes in human miRNA expression can regulate viral replication and persistence in host tissue and modulate host immune response as well (29). Our research is a pioneering one investigating the miRNA signature of NSPCs upon flaviviral infection.

Our data emphasize the downregulation of four miRNAs in the hNS1 cell line and primary neural precursor cells generated from human fetuses. These four miRNAs are extensively reported to play a vital role in neuropathology or host-virus interaction. miR-9 is a highly abundant miRNA in the vertebrate brain, and it affects neurogenesis, neuronal proliferation, differentiation, and migration (30). miR-9 expression is greatest in the subventricular zone, but it is also expressed in dorsal telencephalon and spinal cord (31, 32). Differential expression of miR-9 is associated with many human neuropathological conditions. In medulloblastoma, reduced miR-9 expression is observed (33). In contrast, higher miR-9 expression is seen in glioblastoma (34). miR-9 also facilitates influenza A virus infection in A549 cells (35). miR-22 is shown to control adult neurogenesis, neuronal migration, and dendritic arborization (36). It is also considered a serum biomarker for hepatitis B virus (HBV) infection (37). miR-124 expression is linked to both pre- and postnatal neuronal differentiation (38). Overexpression of miR-124 suppresses the proliferation of medulloblastoma cells (39). miR-132 enhances HIV replication in Jurkat cells (40). miR-132 is also involved in corneal infection by herpes simplex virus (HSV) (41). In case of influenza A virus infection in lung cells, miR-132 accumulation is observed (42). Our report is the first one showing differential expression of these miRNAs in JEV infection.

miRNAs posttranscriptionally silence gene expression by degrading the mRNAs. These genes are called miRNA target genes, which eventually encode proteins. We wanted to identify if any protein-protein interaction (PPI) exists among the miRNA target genes. PPI networks hold a significant importance in systems biology. Studying a PPI network provides the intricate details of the biological pathways occurring inside a cell. PPI networks are of immense importance in studying disease biology also. Mutation or differential expression or loss of translation of a protein might affect the complex protein network, thus in turn causing functional changes in a biological pathway. A single miRNA controls expression of multiple proteins, which among themselves build up a functional network to maintain cellular homeostasis. Therefore, we were interested in evaluating the target gene (protein) interaction of individual miRNAs. The target gene (protein) networks were built up in miRnet.ca and String database followed by their binarization based on interaction between two genes (proteins). Using community detection algorithms based on iterative greedy optimization methods, we identified different modules among gene-gene interactome networks, and using hub detection and classification techniques borrowed from graph theory, we have further pinpointed key hub genes (namely, two classes, provincial and connector hub genes) which might play crucial roles in the functionality of a given module. High-degree genes in the PPI network made significantly increased contributions to the structural motif of the module and also tended to have high-degree centrality in the interactome network. We separated hub genes into provincial and connector classes and predicted that the two types of hubs had different functional organizations of the remaining network (provincial ones may participate more in within-module information processing while the connector class of hub gene may participate more in between-module information processing). To reliably identify hub genes in PPI networks, we used multiple graph theoretical measures, including vertex degree, motif participation coefficient, and betweenness and closeness centrality (participation coefficient is being reported here). While hubs are most often identified solely on the basis of their high degree, the relationship of degree to other aspects of their topological embedding is less well understood. While clearly interrelated, each of the measures that we apply in this study captures a distinct way in which a community of genes participates in the structure of the whole PPI network.

To test out our prediction about the role of miRNA target gene networks in maintaining the functions of a given module and whether any of the identified genes in a given module showed upregulated or downregulated expression after JEV infection, we subjected the identified class of hub genes in our study to further analysis in TargetScan (http://www.targetscan.org). TargetScan is a web server which shows miRNA binding sites in the target gene. Out of all the hub genes of four miRNAs, we validated the expression of genes which had a binding site for the miRNA as shown in the TargetScan server. Postinfection miRNA upregulation by mimics along with JEV infection downregulates expression of certain hub genes, which eventually emphasizes the key target genes of respective miRs which might play important roles in viral infection in NSPCs. These were ETS1 and IL-6 (miR-9-5p); MAX1, NCOA1, NR3C1, and ESR1 (miR-22-3p); and SDC4, PIK3CA, CAV1, and IQGAP1 (miR-124-3p). miR-132-3p target hub genes have no validated interaction in TargetScan; hence, they were eliminated from validation studies. In contrast, SIRT1 is delineated to be a validated miR-9 target in several publications. But neither in hNS1 nor in hNP cells was its interaction with miR-9 found. SIRT1, apart from being a regulator of NSPC differentiation, takes part in several important biological pathways. SIRT1 is involved in regulating metabolic pathways, stress response, and inflammation. Moreover, cell-type-specific miRNA targets are reported in several publications. One miRNA can target different mRNAs in different cell types and tissues (43, 44). Therefore, we hypothesize that SIRT1 upregulation during JEV infection has a different biological function in NSPCs which is not regulated by miR-9. Notably, all the other validated genes—ETS1 and IL-6 (miR-9-5p); MAX1, NCOA1, NR3C1, and ESR1 (miR-22-3p); and SDC4, PIK3CA, CAV1, and IQGAP1 (miR-124-3p)—belong to the connector class of hub genes, and presumably these connector hubs link multiple modules or communities in the PPI network to one another; thus, their expression in the cell after viral infection might find critical functional roles in JEV pathogenesis as well as maintenance of miRNA machinery.

In order to know the biological function of validated hub genes in flaviviral infection, we performed an extensive literature survey. Apart from IL-6 (miR-9 target), PIK3CA, and CAV1 (miR-124 target), no other target gene has been found to be reported previously either in a case of JEV infection or in infection by any other flaviviruses. However, some of the genes are reported to contribute to neural stem cell proliferation or differentiation. Few genes are shown to modulate hepatitis B virus (HBV), Sendai virus, or Ebola virus infection. ETS1 transcription factor has been reported to play a role in tumor vascularization and invasion (45, 46). In the developing mouse embryo, ETS1 expression is seen in neural tube and neural crest cells (47). ETS1 also regulates radial glial cell formation, which in turn facilitates neuronal progenitor cell migration (48). It also regulates VEGFR3 activation in endothelial cells, thus opening the door for Kaposi’s sarcoma-associated herpesvirus infection (49). In spite of acting as a proinflammatory cytokine, IL-6 has a profound impact on the nervous system. IL-6 promotes STAT-3 activation and in turn regulates astrocytic differentiation (50, 51). Myc-associated factor X (MAX1) belongs to a helix-loop-helix family of transcription factors regulating phases of the cell cycle (52). The NCOA1 gene is also known as steroid receptor coactivator 1 (SRC1). SRC1 regulates neuronal differentiation of stem cells, and this protein is rarely localized in glial lineage cells (53). NCOA1/SRC1 has been associated with HIV genome transcription in human neuroblastoma cell line SHSY-5Y (54). The NR3C1 gene encodes a glucocorticoid receptor which has been reported to regulate hippocampal neurogenesis (55). ESR1 is a transcription factor whose overexpression in neural stem/progenitor cells promotes neuronal and astrocytic differentiation (56). Abnormal expression of ESR1 has been associated with liver disease in HBV infection; specifically, ESR1 polymorphism is associated with HBV-induced liver failure (57). Syndecan 4/SDC4 is a Rig-I interactor protein and regulates Rig-I-mediated antiviral signaling effects after Sendai virus infection (58) in HeLa and HEK293 cells. The PIK3CA gene encodes a catalytic subunit of phosphatidylinositol 3-kinase (PI3K) protein. The PI3K/Akt pathway is responsible for cell survival, and activation of this pathway in neural stem cells is reported to be activated after cerebral infarction (59). Flaviviruses like dengue virus and JEV are known activators of the PI3K/Akt pathway, and thus, they inhibit early apoptosis of the host cells (60). Activation of the said pathway in lipid rafts of NSPCs has been also observed in cases of JEV infection as well (61). Caveolin 1 (Cav1) has been extensively studied in cases of JEV infection, and several reports indicate caveolin-mediated viral entry in neuronal cells (62, 63). In published literature, IQGAP1 has been described as a marker of amplifying neural progenitors in the mouse brain. Upon the vascular endothelial growth factor (VEGF) signaling pathway activation, IQGAP1 aids migration of the neural progenitors (64). IQGAP1 is also involved in actin cytoskeletal remodeling during Ebola virus infection (65). Despite having the mentioned biological roles, whether the genes regulate any common signaling pathway in NSPCs is not reported, hence warranting further research.

miRNAs are also being used for diagnosis and prevention of many infectious diseases and thus have promising clinical applications. For example, inhibition of miR-146a in enterovirus-71-infected mice using antagomiR-146a injection significantly improved survival and can be used as a preventive therapeutic (16). Anti-miR-122 (Miravirsen) is used as a therapy in HCV-infected patients and is already in a phase II clinical trial (66). An miRNA-dependent virus attenuation and vaccine development approach has been employed for both flaviviruses and retroviruses. Insertion of miR-9, miR-124a, miR-128a, and let-7c target sequence in the 3′ noncoding region of TBEV/DEN4 (chimeric neurovirulent tick-borne encephalitis virus [TBEV]/dengue virus containing structural proteins of TBEV) has been shown to prevent lethal viral infection (67). miRNAs are also used as adjuvants to regulate host protein expression that inhibits vaccine efficacy. Endoplasmic reticulum protein PERK initiates apoptosis after viral infection and hampers DNA vaccine response. A plasmid-based HIV vaccine has been developed which carries the HIV envelope gene and an miRNA inhibiting PERK. When BALB/c mice are vaccinated with this plasmid, it enhances the CD8^+^ T-cell-specific response, thus inhibiting HIV replication (68). A very recent study used miR-124 target sequence in the 3′ UTR of West Nile virus, which could block West Nile virus replication specifically in neurons, thus inhibiting encephalitis and inducing a humoral immune response (69). As miR-9 and miR-124 have been already in use as therapeutics during viral infection, we strongly feel that miRNAs identified in our study might be used as therapeutic interventions to protect neural stem/progenitor cells upon flaviviral infection.

In summary, we hereby characterize the neural stem/progenitor cell-specific miRNA profile after JEV infection. These miRNAs might be considered attractive therapeutic targets against JEV infection. In future, we aim to evaluate the intricate molecular regulation of the hub genes of miRNA targets in order to identify their role in flaviviral infection.

## MATERIALS AND METHODS

### Cell culture.

**(i) hNS1 culture.** hNS1 cells, an embryonic forebrain-derived neural stem cell line, are a kind gift from Alberto Martínez-Serrano, Centre of Molecular Biology Severo Ochoa, Autonomous University of Madrid, Madrid, Spain. hNS1 cells (formerly called HNSC.100, a model cell line of hNSCs) were grown according to a previously used protocol (1). Briefly, cells were cultured in poly-d-lysine (Sigma)-coated flasks in Dulbecco's modified Eagle medium (DMEM)–F-12 medium (Invitrogen, CA, USA) containing 20 ng/ml each of recombinant human epidermal growth factor (EGF) and fibroblast growth factor (FGF) (R&D Systems), 1% bovine serum albumin (BSA) (Sigma), gentamicin, and 1× N2 supplement (Invitrogen). Upon 65% confluence, cells were treated with JEV at an MOI of 5 and cells were harvested at 72 h postinfection, a time which has been previously reported to show significant viral infection (1). Mock infection was done by adding the same amount of medium used for JEV infection but without virus.

Cell line authentication has been performed recently using the multiplex short tandem repeat (STR) system. The STR profiles verified were D5S818, D7S820, D16S539, TPOX, vWA, PentaE, and amelogenin.

**(ii) hNPC culture.** Isolation of human neural precursor (hNP) cells from aborted human fetuses was carried out according to the ethical guidelines of the National Brain Research Centre (NBRC), the Department of Biotechnology (DBT), and the Indian Council of Medical Research (ICMR) for Stem Cell Research. Briefly, cells were cultured in poly-d-lysine (Sigma)-coated flasks in neurobasal medium (Invitrogen) containing neural survival factor (Lonza), N2 supplement (Invitrogen), 25 ng/ml bovine fibroblast growth factor (bFGF) (Sigma-Aldrich), and 20 ng/ml epidermal growth factor (EGF) (Sigma-Aldrich). Upon 65% confluence, cells were treated with JEV at an MOI of 5 and cells were harvested at 72 h postinfection. JEV infection in hNP cells was found to be the highest at 72 h. Mock infection was done by adding the same amount of medium used for JEV infection but without virus.

### JEV infection in cells.

Both hNS1 and hNP cells were infected with JEV at an MOI of 5 for 72 h and harvested for infection studies, miRNA isolation, and RNA isolation. Control cells were treated with an equal volume of PBS.

### microRNA array.

miRNA isolation from hNS1 cells and fetal neural stem cells was performed using the miRNeasy minikit (Qiagen, CA, USA) according to the manufacturer’s instructions. cDNA preparation was done using the miScript II RT kit (Qiagen). The reaction conditions were 37°C for 60 min and 95°C for 5 min. The miScript miRNA PCR array assay of human neurological development and disease was performed using the miScript SYBR green PCR kit (Qiagen). The PCR array contains 84 miRNAs which are differentially expressed during neuronal development and are responsible for progression of neurological diseases.

### Real-time PCR of miRNA.

To check the expression of hsa-miR-9-5p, hsa-miR-22-3p, hsa-miR-124-3p, and hsa-miR-132-3p, miRNA isolation was performed from harvested control and JEV-infected hNS1 and hNP cells. cDNA was prepared using the miScript II RT kit (Qiagen) as mentioned above. Primers for respective miRNAs (Qiagen) were used in qRT-PCRs and are as follows: miR-9-5p, 5′-UCUUUGGUUAUCUAGCUGUAUGA-3′; miR-22-3p, 5′-AAGCUGCCAGUUGAAGAACUGU-3′; miR-124-3p, 5′-UAAGGCACGCGGUGAAUGCC-3′; and miR-132-3p, 5′-UAACAGUCUACAGCCAUGGUGC-3′. The miScript SYBR green PCR kit (Qiagen) was used for all qRT-PCRs. The reaction conditions for qRT-PCR were 95°C for 15 min (1 cycle) and 40 cycles of 94°C for 15 s, 55°C for 30 s, and 70°C for 30 s. SNORD68 was used as an internal control. Data analysis was done using the comparative threshold cycle (ΔΔ*C_T_*) method.

### Transfection of cells with miRNA mimic and inhibitor.

To overexpress or to reduce miR-9-5p, miR-22-3p, miR-124-3p, and miR-132-3p expression in hNS1 and hNP cells, transfection was done with human miRNA mimics (double-stranded RNAs that mimic mature endogenous miRNAs, procured from Exiqon) or inhibitors (single-stranded modified RNAs [Exiqon]) as mentioned above. HiPerFect transfection reagent (Qiagen) was used according to the manufacturer’s protocol. At 24 h posttransfection, either cells were collected to check the transfection efficiency, or they were infected with JEV for specific time points and harvested accordingly. Controls of the mimic (Ambion) and inhibitors (Exiqon) were used in all transfection experiments. Mock transfection (control/mock) cells received an equal volume of HiPerFect reagent without any nucleic acids.

### RNA isolation and real-time PCR for target genes.

hNS1 and hNP cells were harvested from culture plates using TRIzol reagent (Sigma, USA) followed by chloroform treatment. These samples were centrifuged at 12,000 rpm for 15 min at 4°C for phase separation. Aqueous phase was carefully collected, and isopropanol was added to it, followed by centrifugation at 12,000 rpm for 15 min at 4°C to get the RNA pellet. The RNA pellet was washed in 75% ethanol and air dried. cDNA was synthesized with 100 ng RNA using the Advantage RT-PCR kit (Clontech, Mountain View, CA, USA). The reaction conditions for real-time PCR were 95°C for 3 min (1 cycle) and 95°C for 30 s, annealing temperature for 30 s, and 72°C for 30 s (45 cycles). The results were normalized using human GAPDH by the ΔΔ*C_T_* method and represented as fold change. Primer sequences are listed in Table S1 in the supplemental material.

10.1128/mSphere.00588-19.7 TABLE S1 Primer sequences (human) used in qRT-PCRs. Download Table S1, PDF file, 0.1 MB. Copyright © 2019 Mukherjee et al. 2019 Mukherjee et al. This content is distributed under the terms of the Creative Commons Attribution 4.0 International license.

10.1128/mSphere.00588-19.8 TABLE S2 miRNA fold change profile of hNS1 cells. Download Table S2, PDF file, 0.1 MB. Copyright © 2019 Mukherjee et al. 2019 Mukherjee et al. This content is distributed under the terms of the Creative Commons Attribution 4.0 International license.

10.1128/mSphere.00588-19.9 TABLE S3 miRNA fold change profile of hNP cells. Download Table S3, PDF file, 0.1 MB. Copyright © 2019 Mukherjee et al. 2019 Mukherjee et al. This content is distributed under the terms of the Creative Commons Attribution 4.0 International license.

10.1128/mSphere.00588-19.10 TABLE S4 miRNA target genes and their repetition frequency in biological pathways. Download Table S4, PDF file, 0.1 MB. Copyright © 2019 Mukherjee et al. 2019 Mukherjee et al. This content is distributed under the terms of the Creative Commons Attribution 4.0 International license.

### microRNA isolation from human autopsy tissue.

miRNA was isolated from paraffin-embedded basal ganglion tissue of human autopsy cases validated to have JEV infection (cerebrospinal fluid [CSF] positive for IgM). Control tissue was collected from subjects who died due to road accidents and did not have a prior record of infection in the brain. The tissue samples were collected from the Human Brain Bank, NIMHANS, Bangalore, India, according to institutional ethical rules. Deparaffinization was carried out according to our previously published article (70). Then, the tissue samples were homogenized using Qiazol reagent (Qiagen), and the rest of the isolation procedure was performed using the miRNeasy minikit (Qiagen).

### Graph theoretic analysis of miRNA target gene PPI networks.

Graphs are composed of vertices (or nodes; here equivalent to target genes) and edges (or connections; here equivalent to intergene connectivity patterns). The connectivity structure of a graph is represented typically by its adjacency matrix; here, we first construct an asymmetric binary matrix representing directed but unweighted edges. Paths are ordered sequences of edges linking pairs of vertices (a source and a target). The distance between two vertices corresponds to the length (number of edges) of the shortest path between them. The distance matrix of a graph comprises all pairwise distances. Its maximum corresponds to the graph diameter, its minimum corresponds to the graph radius, and its average corresponds to the graph’s characteristic path length.

Basic graph measures such as connection density, proportion of reciprocal connections, degree distributions, measures derived from the distance matrix (diameter, radius, and path length), and participation coefficients were calculated using standard graph theory methods. Brain Connectivity Toolbox (BCT) in Matlab was used to compute modularity score and the participation coefficient of all the four miRNA target genes (71).

### Community structure identification in target gene PPI networks.

Modularity score is used to measure the community structure within a network. The value of modularity ranges between [−0.5, 1] with 0 and negative values meaning a network with randomly assigned edges and positive values indicating a highly communal structure. In a given graph G (V, E) which can be partitioned into two membership variables *s*, if a node *i* falls into community 1, then *s_i_* = 1 or else *s_i_* = −1. An adjacency matrix may be denoted by *A*, which says that *A_ij_* = 1 means that there is a connection between nodes *i* and *j* and that *A_ij_* = 0 when there are no interactions. Modularity (*Q*) is then defined as the fraction of edges that fall within community 1 or 2, minus the expected number of edges within communities 1 and 2 for a random graph with the identical node degree distribution as the given graph. All the modularity analysis is carried out by comparisons against populations of degree-matched random networks.

To identify modules (communities) within each target gene network, we apply a variant of a spectral community detection algorithm. Formally, modularity (*Q*) can be defined as

(1) $$Q=\sum_{c_{i\in C}}\left[\frac{\left|E_{c_{i}}^{\text{in}}\right|}{\left|E\right|}-{\left(\frac{2|E_{c_{i}}^{\text{in}}\left|+|E_{c_{i}}^{\text{out}}\right|}{\left|E\right|}\right)}^{2}\right]$$

where *C* is the set of all miRNA target gene communities, *c_i_* is a specific community in *C*, $\left|E_{c_{i}}^{\text{in}}\right|$ is the number of edges between node genes within community *c_i_*, $\left|E_{c_{i}}^{\text{out}}\right|$ is the number of edges from genes in community *c_i_* to target genes outside community *c_i_*, and |*E*| is the total number of edges in the gene network. For simplicity, modularity in equation 1 can also be expressed in a more compact form:

(2) $$Q=\frac{1}{2\left|E\right|}\sum_{\mathrm{ij}}\left[A_{\mathrm{ij}}-\frac{k_{i}k_{j}}{2\left|E\right|}\right]\text{δ}c_{i},c_{j}$$

where *k_i_* is the degree of node *i*, *A_ij_* is an element of the adjacency matrix, δ_*c*_*i*_,*c*_*i*__ is the Kronecker delta symbol, and *c_i_* is the label of the community to which a target gene node *i* is being assigned. The modularity measure defined above is suitable for only undirected and unweighted networks. However, this definition can be naturally extended to apply to directed networks as well as to weighted networks. Weighted and directed networks contain more information than undirected and unweighted ones and are therefore often usefully viewed as more valuable but also as more difficult to analyze than their simpler counterparts. The revised definition of modularity that works for directed networks is as follows:

(3) $$Q=\frac{1}{\left|E\right|}\sum_{\mathrm{ij}}\left[A_{\mathrm{ij}}-\frac{k_{i}^{\text{in}}k_{j}^{\text{out}}}{\left|E\right|}\right]{\text{δ}}_{c_{i},c_{j}}$$

where $k_{i}^{\text{in}}$ and $k_{i}^{\text{out}}$ are the in and out degrees of the gene network. As inputs to the algorithm, we used matrices of matching indices (25), which express the similarity of connection patterns for each pair of vertices. Once modules were detected, different solutions were ranked according to a cost function and the optimal modularity (out of 10,000 solutions for a range of between 2 and 6 modules) was used as the basis for hub classification. Details of the modularity optimization techniques used are outlined below.

### Modularity optimization of identified community structure spectral methods and greedy search.

Modularity in equation 3 can be rewritten as

(4) $$Q=\frac{1}{4\left|E\right|}\sum_{\mathrm{ij}}\left[A_{\mathrm{ij}}-\frac{k_{i}k_{j}}{2\left|E\right|}\right]\left(s_{i}s_{j}+1\right)$$

where *s* is column vector representing any division of the network into two groups. The elements of the column vector are defined as *s_i_* = +1 if node *i* belongs to the first group and *s_i_* = −1 if the node belongs to the second group. The modularity matrix *B* with elements can be written as

(5) $$B_{\mathrm{ij}}=A_{\mathrm{ij}}-\frac{k_{i}k_{j}}{2\left|E\right|}$$

Representing the column vector *s* as a linear combination of the normalized eigenvectors *u_i_* of modularity matrix *B*, $s=\sum_{i=1}^{\left|V\right|}a_{i}u_{i}$ with $a_{i}=u_{i}^{\text{T}}.s$, and then plugging the result into equation 5 yields

(6) $$Q=\frac{1}{4\left|E\right|}\sum_{i}a_{i}u_{i}^{\text{T}}.B\sum_{j}a_{j}u_{j}=\frac{1}{4\left|E\right|}\sum a_{i}^{2}{\text{β}}_{i}$$

where β*_i_* is the eigenvalue of *B* corresponding to eigenvector *u_i_*. To maximize *Q* above, Newman (72) proposed a spectral approach to choose *s* proportional to the leading eigenvector *u*_1_ corresponding to the largest (most positive) eigenvalue β*_i_*. The algorithm that we used initially divided the network into two communities, and in further iterations the community structure is subdivided. The choice assumes that the eigenvalues are labeled in decreasing order ${\text{β}}_{1}\geq {\text{β}}_{2}\geq \ldots \geq {\text{β}}_{\left|V\right|}$. Nodes are then divided into two communities according to the signs of the elements in *s* with nodes corresponding to positive elements in *s* assigned to one group and all remaining nodes corresponding to another. Since the row and column sums of *B* are zero, it always has an eigenvector (1, 1, 1, …) with eigenvalue zero. Therefore, if it has no positive eigenvalue, then the leading eigenvector is (1, 1, 1, …), which means that the network is indivisible. Once *Q* is almost 0 for an indivisible network, then further subdividing beyond this point will not contribute to the increase in modularity value *Q*. This can be used to terminate community structure division. Hence, the algorithm would end at a certain point when the optimal network has been estimated. In order to fine-tune this method of community structure optimization further, this fitness function is typically performed using the Louvain method (72), a greedy agglomerative clustering algorithm that works on hierarchical refinements of the network’s partitions. Here, we used the Louvain implementation available in the Brain Connectivity Toolbox (71).

### Classification of miRNA target hub genes.

To classify hub genes in the community which are potential miRNA targets, we adopted the following strategy. First, we calculated each vertex’s participation index *P* (73), which expresses its distribution of intra- versus intermodule connections. *P* of vertex *v* is defined as

(7) $$P_{v}=1-\sum_{s=1}^{N_{M}}{\left(\frac{k_{\mathrm{vs}}}{k_{v}}\right)}^{2}$$

where *N_M_* is the number of identified modules, *k_v_* is the degree of node *v*, and *k_vs_* is the number of edges from the *v*th node to nodes within module *s*. Considering only high-degree vertices (i.e., vertices with a degree at least 1 standard deviation [SD] above the network mean), we classify vertices with a participation coefficient *P < *0.4 as provincial hubs and nodes with *P* > 0.4 as connector hubs. Since *P* cannot exceed 0.5 for two-module networks and 0.67 for three-module networks, kinless hubs (i.e., nodes with *P* > 0.8) cannot occur in these gene-gene interactome networks.

### Statistical analysis.

Data are represented as mean ± SD from three independent experiments (*n* = 3). Statistical significance was calculated using Student’s *t* test in the case of two experimental groups or one-way analysis of variance (ANOVA) for multiple groups followed by Bonferroni *post hoc* test. A *P* value of <0.05 was considered to be statistically significant.
