# Supplementary figures and images for: Identification and Classification of Hubs in microRNA Target Gene Networks in Human Neural Stem/Progenitor Cells following Japanese Encephalitis Virus Infection
Source: mSphere. 2019 Oct 2;4(5):e00588-19. doi: 10.1128/mSphere.00588-19 (PMC6796970; doi:10.1128/mSphere.00588-19)

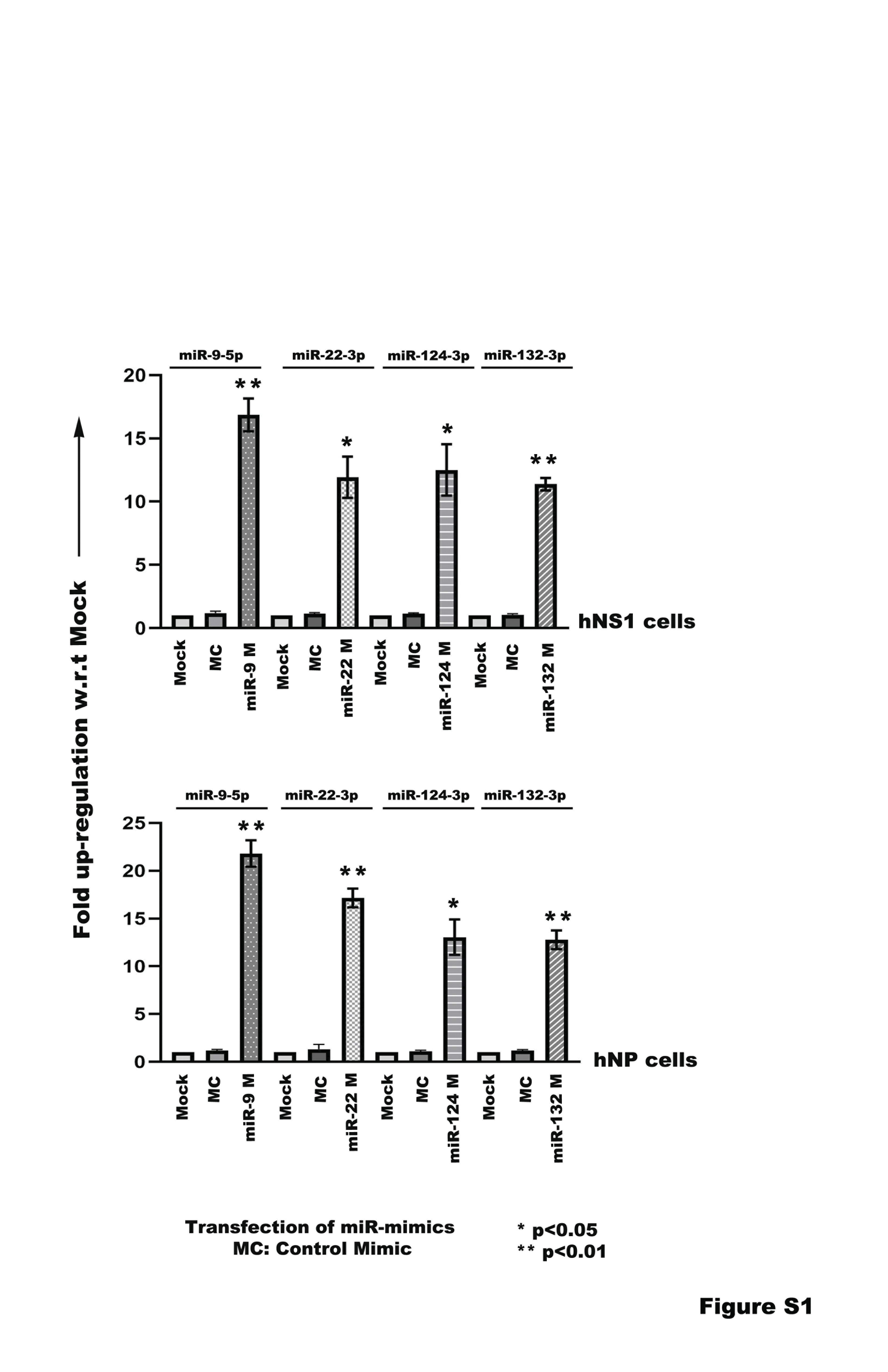

Supplement: FIG S1 [file mSphere.00588-19-sf001.tif]

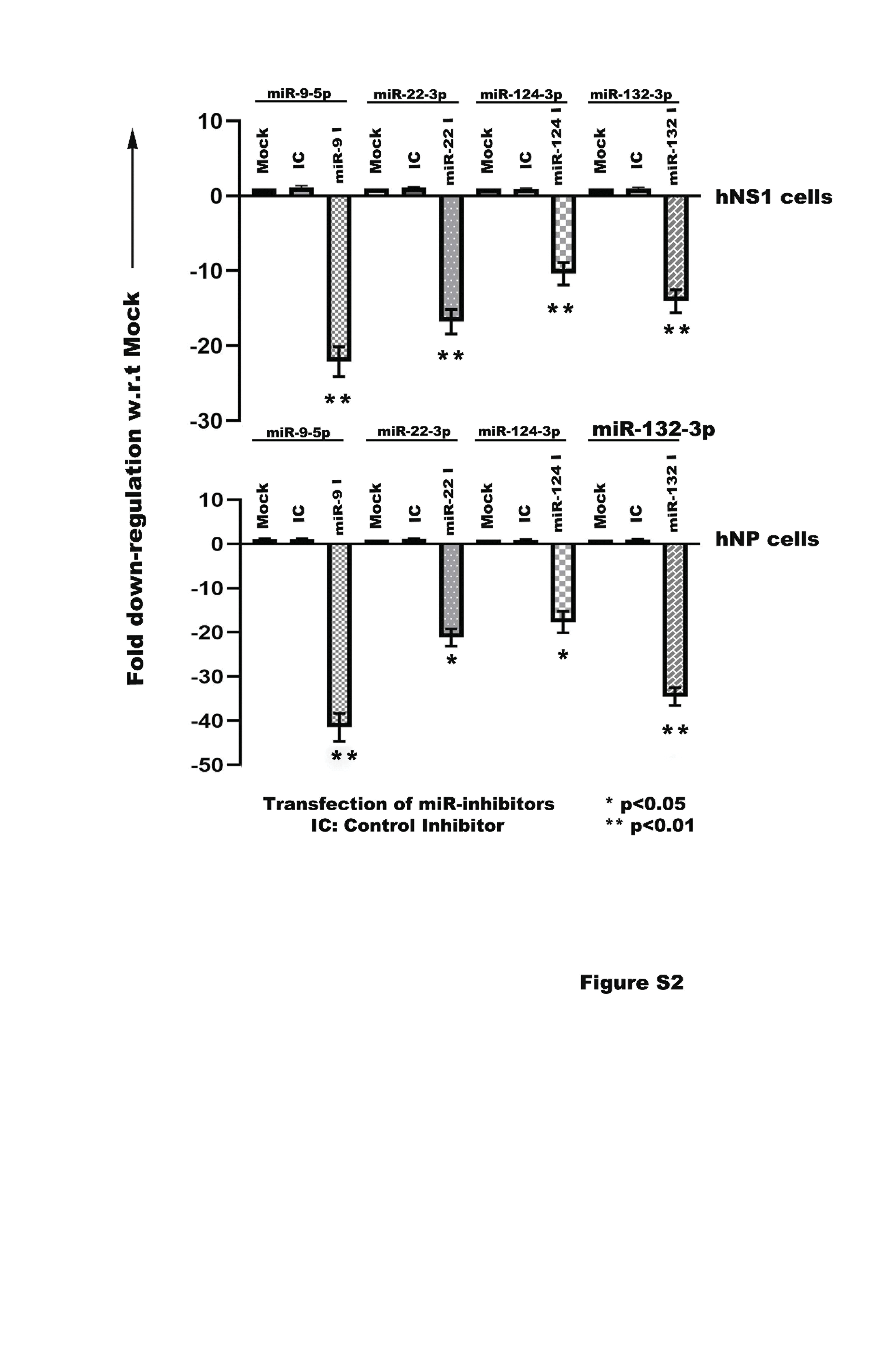

Supplement: FIG S2 [file mSphere.00588-19-sf002.tif]

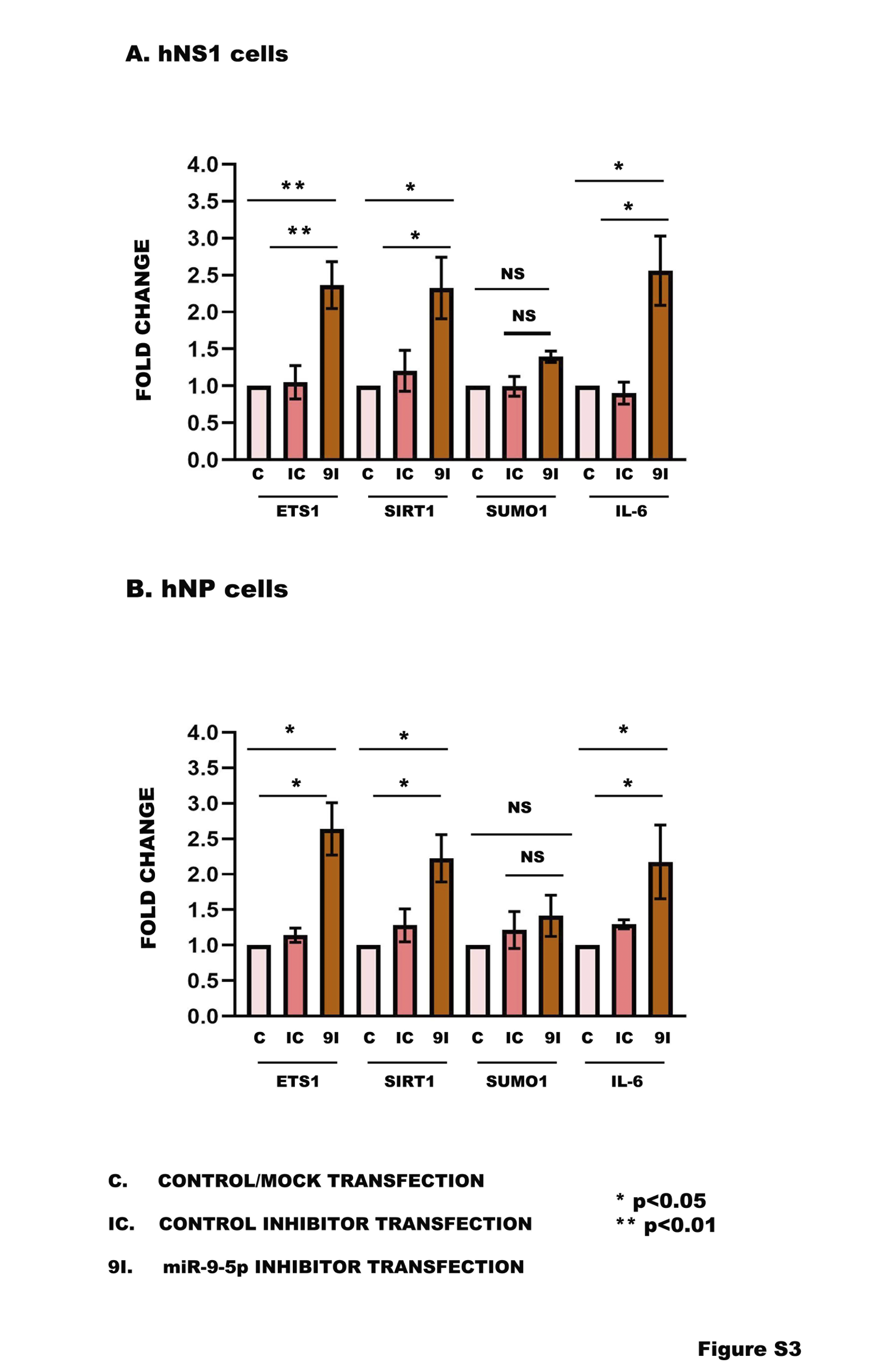

Supplement: FIG S3 [file mSphere.00588-19-sf003.tif]

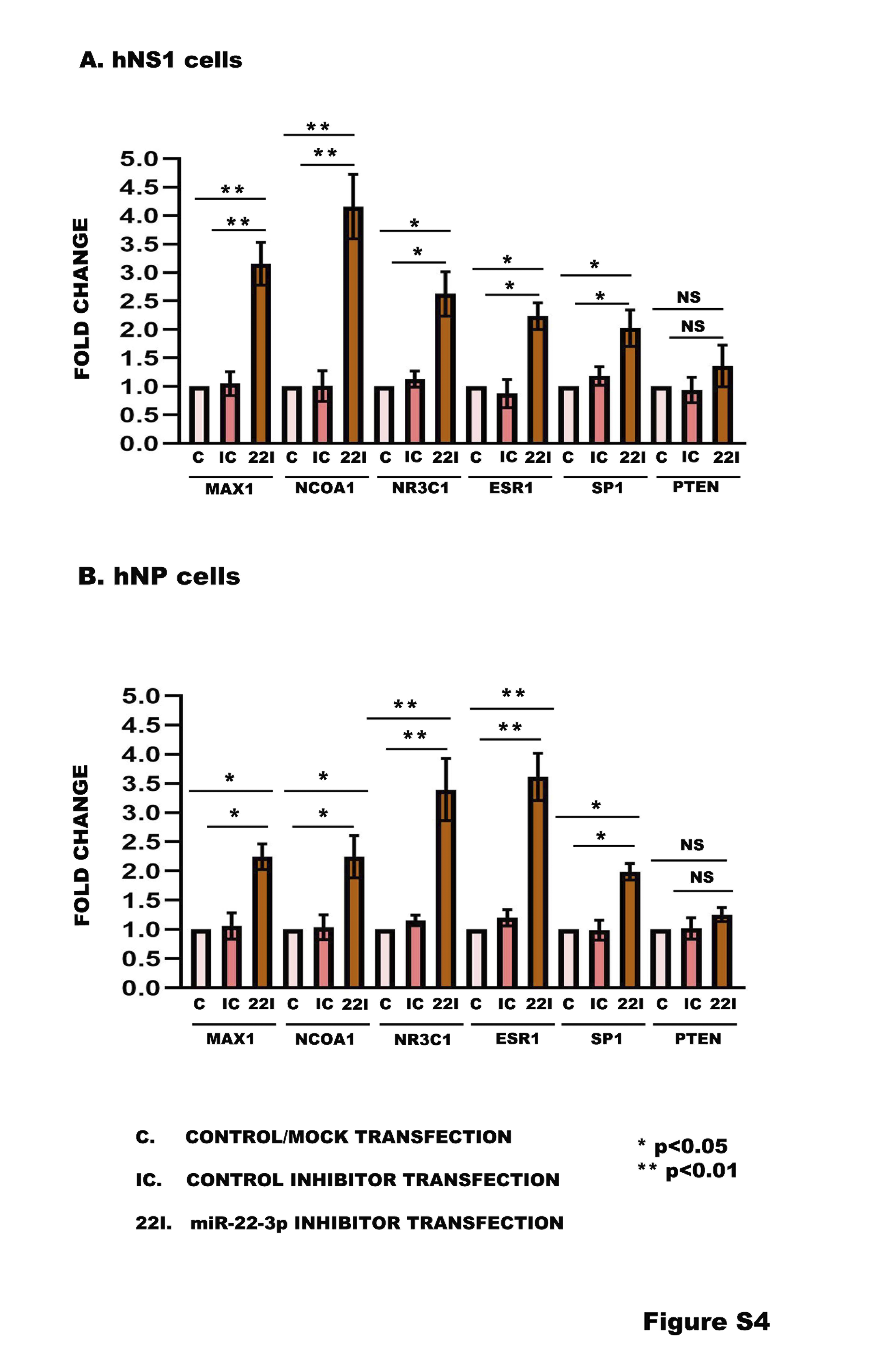

Supplement: FIG S4 [file mSphere.00588-19-sf004.tif]

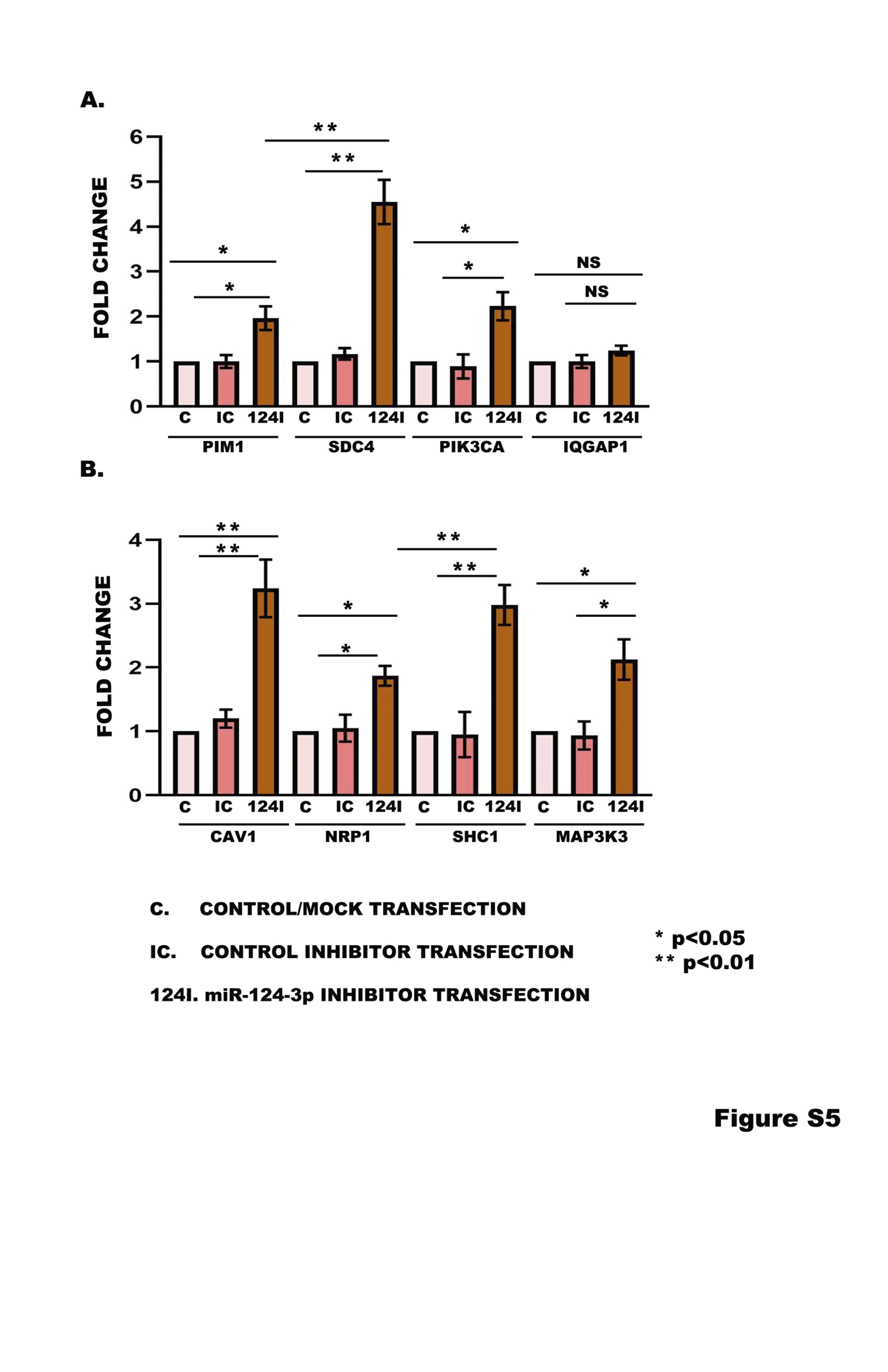

Supplement: FIG S5 [file mSphere.00588-19-sf005.tif]

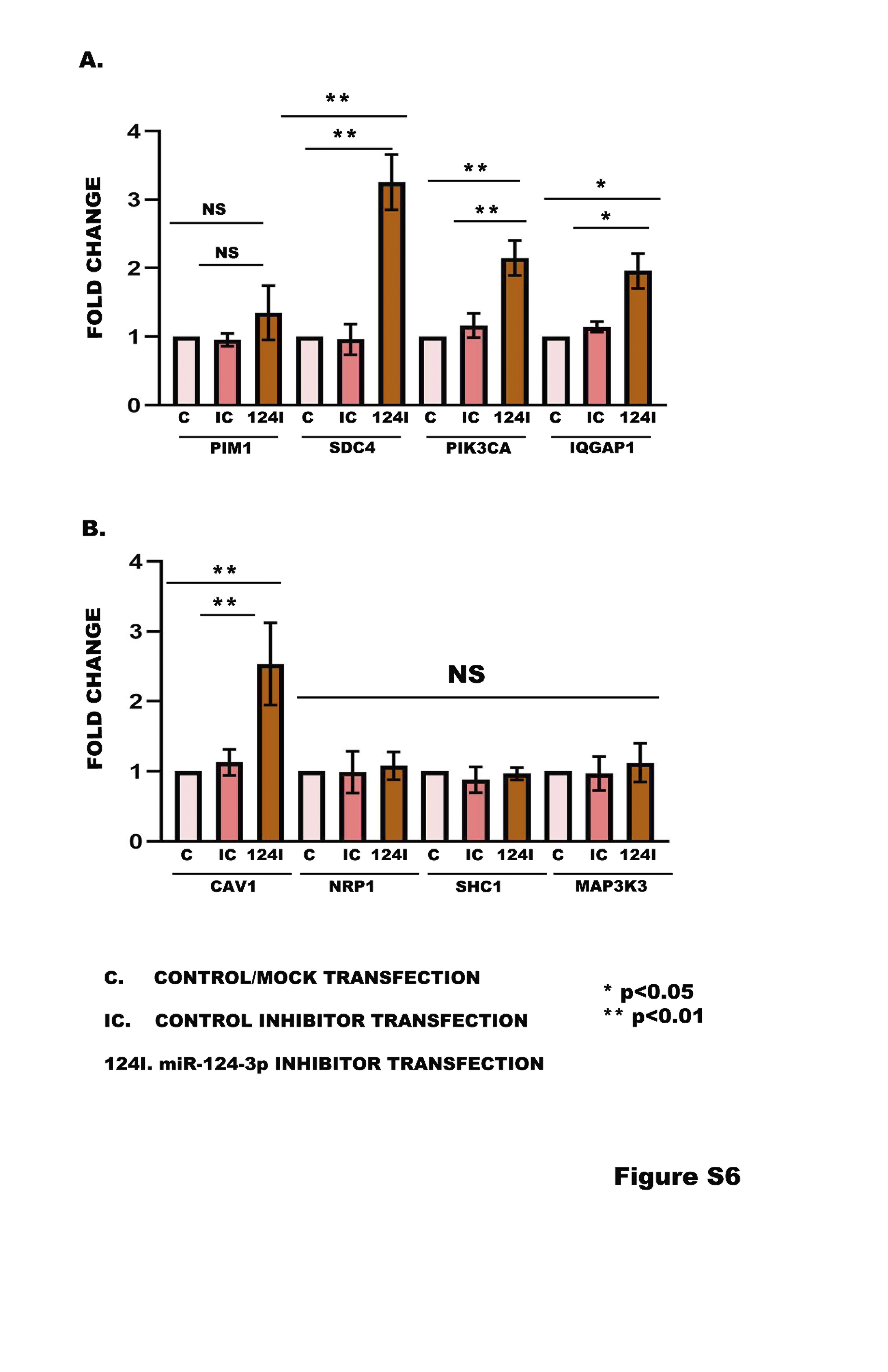

Supplement: FIG S6 [file mSphere.00588-19-sf006.tif]
