# Supplementary material for: Identification and Classification of Hubs in microRNA Target Gene Networks in Human Neural Stem/Progenitor Cells following Japanese Encephalitis Virus Infection
Source: mSphere. 2019 Oct 2;4(5):e00588-19. doi: 10.1128/mSphere.00588-19 (PMC6796970; doi:10.1128/mSphere.00588-19)
Supplement: TABLE S1 [file mSphere.00588-19-st001.pdf]

Name of Primer (Human)	Forward / Reverse	Sequence(5’– 3’)
NRP1	F	TTCATTGCTCGTTCCCCTCC
	R	TCGCCTGCATCCTGTCATTT
CAV1	F	GGAGTGTCCGCTTCTGCTAT
	R	GCCCAACAGACCCAATCTCA
IQGAP1	F	GATCTGCCCCTCCTATCTGTC
	R	CCCCAGTTTGGCAAGGTAGA
MAP3K3	F	ATCCTTGGACAGGTCAGCAG
	R	TCCAGGTATTGCACAGCCAG
PIM	F	AAGGACCGGATTTCCGACTC
	R	AAGAGATCTTGCACCGGCTC
SDC4	F	ACTTTGAGCTGTCTGGCTCTG
	R	AGTTTCTTGGGTTCGGTGGG
PIK3CA	F	AAATGCTTGGGGTGGAAGGG
	R	ATGTATTCAGTTCAATTGCAGAAGG
SHC1	F	CTCCCCAGGACTTCTGTGACT
	R	GGGGGCAGGAGATCCATAGT
PTEN	F	TGTAGTAAGTTGTGCTGAAAGACA
	R	CACCAGTTCGTCCCTTTCCA
NR3C1A	F	GCAGTGGAAGGACAGCACAA
	R	CTCCAACAGTGACACCAGGG
ESR1	F	TGCGTCGCCTCTAACCTCG
	R	TCCCAGATGCTTTGGTGTGG
NCOA1A	F	TCTGCATCTCACCACCCCTA
	R	ACTCGTTAATTTGGGCTTTGGC
MAX1	F	TCACAGTTTGCGGGACTCAG
	R	GCACATACTCCATGACTGGC
SP1	F	CGCCCTCTGACCAAGATCACT
	R	GGGAGTTGTTGCTGTTCTCATTGG
SUMO1	F	TCAACTGAGGACTTGGGGGA
	R	CAAAGAGATGGGGTGCCAGT
ETS1	F	AAAGGCAGCGGGAATTTGAG
	R	AAGCAGTCTTTACCCAGGGC
IL-6	F	CCAGCTATGAACTCCTTCTC
	R	GCTTGTTCCTCACATCTCTC
SIRT1	F	ACAGGTTGCGGGAATCCAA
	R	GGCAAGATGCTGTTGCAAA
