# Supplementary material for: Identification and Classification of Hubs in microRNA Target Gene Networks in Human Neural Stem/Progenitor Cells following Japanese Encephalitis Virus Infection
Source: mSphere. 2019 Oct 2;4(5):e00588-19. doi: 10.1128/mSphere.00588-19 (PMC6796970; doi:10.1128/mSphere.00588-19)
Supplement: TABLE S2 [file mSphere.00588-19-st002.pdf]

Layout	**01**	**02**	**03**	**04**	**05**	**06**	**07**	**08**	**09**	**10**	**11**	**12**
**A**	hsa-let-7b-5p **1.15**	hsa-let-7c-5p **-1.02**	hsa-let-7d-5p **-1.05**	hsa-let-7e-5p **1.27**	hsa-let-7i-5p **1.56**	hsa-miR-101-3p **-2.49**	hsa-miR-105-5p **1.15**	hsa-miR-106b-5p **1.27**	hsa-miR-107 **-1.17**	hsa-miR-124-3p **-12.35**	hsa-miR-125b-5p **1.03**	hsa-miR-126-5p **-12.42**
**B**	hsa-miR-128-3p **-5.79**	hsa-miR-130a-3p **-1.03**	hsa-miR-132-3p **-4.11**	hsa-miR-133b **-3.67**	hsa-miR-134-5p **-2.44**	hsa-miR-135b-5p **1.59**	hsa-miR-138-5p **-15.67**	hsa-miR-139-5p **-8.06**	hsa-miR-140-5p **-1.04**	hsa-miR-146a-5p **-2.13**	hsa-miR-146b-5p **1.55**	hsa-miR-148b-3p **1.18**
**C**	hsa-miR-151a-3p **1.74**	hsa-miR-152-3p **-1.88**	hsa-miR-15a-5p **-1.01**	hsa-miR-15b-5p **1.56**	hsa-miR-181a-5p **1.11**	hsa-miR-181d-5p **1.31**	hsa-miR-191-5p **1.01**	hsa-miR-193b-3p **1.26**	hsa-miR-195-5p **1.08**	hsa-miR-19b-3p **-1.11**	hsa-miR-203a-3p **-2.32**	hsa-miR-20a-5p **1.35**
**D**	hsa-miR-212-3p **-1.31**	hsa-miR-22-3p **-4.58**	hsa-miR-24-3p **1.16**	hsa-miR-26b-5p **-1.07**	hsa-miR-27a-3p **1.12**	hsa-miR-28-5p **1.88**	hsa-miR-298 **1.15**	hsa-miR-29a-3p **-2.60**	hsa-miR-29b-3p **-10.74**	hsa-miR-29c-3p **-3.33**	hsa-miR-302a-5p **1.15**	hsa-miR-302b-5p **1.15**
**E**	hsa-miR-30d-5p **-1.18**	hsa-miR-320a **1.64**	hsa-miR-328-3p **-1.92**	hsa-miR-337-3p **2.24**	hsa-miR-338-3p **-13.69**	hsa-miR-339-5p **1.08**	hsa-miR-342-3p **-2.02**	hsa-miR-346 **-2.68**	hsa-miR-34a-5p **-1.11**	hsa-miR-376b-3p **1.16**	hsa-miR-381-3p **-9.33**	hsa-miR-409-3p **-1.15**
**F**	hsa-miR-431-5p **-2.45**	hsa-miR-432-5p **2.00**	hsa-miR-433-3p **-6.71**	hsa-miR-455-5p **1.24**	hsa-miR-484 **1.21**	hsa-miR-485-3p **-1.58**	hsa-miR-485-5p **-2.54**	hsa-miR-487a-3p **2.53**	hsa-miR-488-3p **-1.01**	hsa-miR-489-3p **1.12**	hsa-miR-499a-5p **1.15**	hsa-miR-509-3p **1.00**
**G**	hsa-miR-511-5p **1.15**	hsa-miR-512-3p **-1.04**	hsa-miR-518b **1.15**	hsa-miR-539-5p **-2.64**	hsa-miR-652-3p **-1.39**	hsa-miR-7-5p **-6.03**	hsa-miR-9-5p **-2.50**	hsa-miR-9-3p **-2.75**	hsa-miR-92a-3p **1.56**	hsa-miR-93-5p **1.60**	hsa-miR-95-3p **1.84**	hsa-miR-98-5p **1.64**
**H**	cel-miR-39-3p **1.15**	cel-miR-39-3p **1.15**	SNORD61 **1.06**	SNORD68 **-1.24**	SNORD72 **-2.49**	SNORD95 **1.30**	SNORD96A **-1.88**	RNU6-6P **1.63**	miRTC **1.06**	miRTC **-1.41**	PPC **1.17**	PPC **1.20**
