# Supplementary material for: Identification and Classification of Hubs in microRNA Target Gene Networks in Human Neural Stem/Progenitor Cells following Japanese Encephalitis Virus Infection
Source: mSphere. 2019 Oct 2;4(5):e00588-19. doi: 10.1128/mSphere.00588-19 (PMC6796970; doi:10.1128/mSphere.00588-19)
Supplement: TABLE S3 [file mSphere.00588-19-st003.pdf]

Layout	**01**	**02**	**03**	**04**	**05**	**06**	**07**	**08**	**09**	**10**	**11**	**12**
**A**	hsa-let-7b-5p **1.01**	hsa-let-7c-5p **-1.38**	hsa-let-7d-5p **-1.27**	hsa-let-7e-5p **-1.29**	hsa-let-7i-5p **-1.33**	hsa-miR-101-3p **1.00**	hsa-miR-105-5p **1.01**	hsa-miR-106b-5p **-1.43**	hsa-miR-107 **-1.96**	hsa-miR-124-3p **-4.16**	hsa-miR-125b-5p **-1.24**	hsa-miR-126-5p **-2.03**
**B**	hsa-miR-128-3p **-1.79**	hsa-miR-130a-3p **-1.18**	hsa-miR-132-3p **-2.41**	hsa-miR-133b **1.01**	hsa-miR-134-5p **1.03**	hsa-miR-135b-5p **-1.48**	hsa-miR-138-5p **-2.26**	hsa-miR-139-5p **-2.23**	hsa-miR-140-5p **-1.29**	hsa-miR-146a-5p **1.11**	hsa-miR-146b-5p **1.16**	hsa-miR-148b-3p **-1.54**
**C**	hsa-miR-151a-3p **-1.36**	hsa-miR-152-3p **-2.41**	hsa-miR-15a-5p **-1.25**	hsa-miR-15b-5p **-1.46**	hsa-miR-181a-5p **-1.44**	hsa-miR-181d-5p **-1.49**	hsa-miR-191-5p **-1.34**	hsa-miR-193b-3p **-1.45**	hsa-miR-195-5p **-1.28**	hsa-miR-19b-3p **-1.31**	hsa-miR-203a-3p **1.01**	hsa-miR-20a-5p **-1.50**
**D**	hsa-miR-212-3p **-1.33**	hsa-miR-22-3p **-2.43**	hsa-miR-24-3p **-1.33**	hsa-miR-26b-5p **-1.33**	hsa-miR-27a-3p **-1.17**	hsa-miR-28-5p **-1.27**	hsa-miR-298 **-1.20**	hsa-miR-29a-3p **-1.23**	hsa-miR-29b-3p **-1.07**	hsa-miR-29c-3p **-1.09**	hsa-miR-302a-5p **1.01**	hsa-miR-302b-5p **1.01**
**E**	hsa-miR-30d-5p **-1.28**	hsa-miR-320a **-1.37**	hsa-miR-328-3p **-1.52**	hsa-miR-337-3p **-1.41**	hsa-miR-338-3p **-1.35**	hsa-miR-339-5p **1.19**	hsa-miR-342-3p **-1.43**	hsa-miR-346 **1.16**	hsa-miR-34a-5p **-1.29**	hsa-miR-376b-3p **-1.87**	hsa-miR-381-3p **-1.23**	hsa-miR-409-3p **-1.32**
**F**	hsa-miR-431-5p **1.70**	hsa-miR-432-5p **-1.62**	hsa-miR-433-3p **-1.22**	hsa-miR-455-5p **-1.47**	hsa-miR-484 **-1.30**	hsa-miR-485-3p **1.25**	hsa-miR-485-5p **1.56**	hsa-miR-487a-3p **-1.03**	hsa-miR-488-3p **-1.44**	hsa-miR-489-3p **-1.07**	hsa-miR-499a-5p **-3.05**	hsa-miR-509-3p **1.01**
**G**	hsa-miR-511-5p **1.17**	hsa-miR-512-3p **1.01**	hsa-miR-518b **1.01**	hsa-miR-539-5p **-1.51**	hsa-miR-652-3p **-1.59**	hsa-miR-7-5p **-1.03**	hsa-miR-9-5p **-2.17**	hsa-miR-9-3p **-1.15**	hsa-miR-92a-3p **-1.38**	hsa-miR-93-5p **-1.64**	hsa-miR-95-3p **-1.10**	hsa-miR-98-5p **-1.34**
**H**	cel-miR-39-3p **1.01**	cel-miR-39-3p **1.01**	SNORD61 **-1.12**	SNORD68 **-1.05**	SNORD72 **-1.13**	SNORD95 **1.17**	SNORD96A **1.07**	RNU6-6P **-1.07**	miRTC **1.31**	miRTC **-1.00**	PPC **1.01**	PPC **1.05**
