# Supplementary material for: Identification and Classification of Hubs in microRNA Target Gene Networks in Human Neural Stem/Progenitor Cells following Japanese Encephalitis Virus Infection
Source: mSphere. 2019 Oct 2;4(5):e00588-19. doi: 10.1128/mSphere.00588-19 (PMC6796970; doi:10.1128/mSphere.00588-19)
Supplement: TABLE S4 [file mSphere.00588-19-st004.pdf]

MICRORNA NAME	GENE NAMES	REPETITION NUMBER IN BIOLOGICAL PATHWAYS	GENE NAMES	REPETITION NUMBER IN BIOLOGICAL PATHWAYS	GENE NAMES	REPETITION NUMBER IN BIOLOGICAL PATHWAYS
**hsa-miR-9-5p**	ATG10	2	F2	11	NRP1	20
	ATG5	7	FOXO1	18	NOTCH1	42
	BCL2	34	FOXO3	19	NOTCH2	15
	BCL2L11	15	GDNF	20	NUP155	5
	BCL6	21	GSK3B	32	PDGFRB	18
	BECN1	7	HDAC4	18	POU2F2	5
	CAMKK2	3	HES1	37	POU2F1	5
	CCL19	18	IL5	8	PRKAA1	12
	CCND1	12	IL6	34	PPARA	18
	CD34	15	KPNB1	3	RUNX1	18
	CDH1	18	LRP1	5	SDC1	6
	CREB1	19	MMP9	3	SOD2	13
	CUL4A	2	MMP2	6	SPI1	19
	CXCR4	23	MMP13	2	SRF	29
	DKK1	23	MLL2	14	SRSF1	3
	DRD2	28	MYF6	16	SUMO1	9
	EFNA1	26	MYOCD	24	SIRT1	29
	EP300	20	NCOR2	7	SNAI2	26
	ESR1	13	NFATC3	9	VEGFA	42
	ETS1	23	NFKB1	15	WNT6	11
	ELAVL1	2	NF1	37	WNT8A	12
**hsa-miR-22-3p**	ARPC5	2	ESR1	6	NTRK2	11
	ACVR1C	5	ERBB2	8	PRKACA	5
	ALMS1	7	GRB2	2	PPARA	6
	AKT1	20	HDAC6	4	PLK1	7
	BUB1B	6	HDAC4	6	PTEN	16
	BDNF	7	HMGB1	10	RAB5B	2
	CCNA2	2	HIF1A	13	RBL1	3
	CCNT2	4	LGALS1	3	SP1	4
	CDK6	4	MAX	2	SNAI1	7
	CSF1R	5	MTDH	4	SIRT1	15
	CDKN1A	10	MMP14	5	TPD52L2	2
	DDIT4	6	NUP214	2	YWHAZ	2
	E2F2	2	NR3C1	3		
	ERBB3	4	NCOA1	4		
**hsa-miR-124-3p**	ACTR3	5	FGFR1	16	PIM1	7
	ADCY3	7	GAS6	14	PIK3CA	8
	ADCY6	7	GNAI2	8	PPAP2B	7
	ADCY9	7	GNB2L1	11	PTPN11	10
	ADAM15	5	GRM1	6	PTPRJ	8
	AGTR2	7	GRB2	5	PTGS2	9

	AKT2	7	HMOX1	9	PRKAG1	6
	AR	6	ID2	7	PXN	9
	ARAF	5	IL6	17	RAC1	8
	B4GALT1	6	IL6R	11	RBPJ	8
	BCL6	8	IL11	5	RELA	6
	BDNF	5	IQGAPI	7	RHOA	16
	CAV1	10	ITGB1	16	ROCK1	12
	CCL2	16	ITGB3	13	ROCK2	9
	CCND2	7	JUP	5	RPS6KA4	5
	CDKN1A	9	KLF4	7	RRAS	7
	CDKN2A	13	LAMA1	5	SDC4	9
	CDK6	6	MAP2K3	6	SERPINE1	10
	CDON	7	MAP3K3	6	SHC1	13
	CTNNB1	15	MAP3K4	6	SIRT1	17
	COL1A1	8	MAPK14	15	SNAI2	12
	CSPG4	8	MAPK11	9	SPHK1	12
	CXCL8	12	MAPKAPK2	7	SPRY2	9
	CYR61	16	MAPKAPK3	7	STAT5A	7
	DRD4	6	MDFIC	8	SOX9	14
	EDN1	15	MYH9	5	TLN1	9
	EFNA1	14	NRG1	14	TLR3	8
	EPHA2	7	NRP1	5	WNT4	8
	EREG	12	NRAS	11	WNT5B	5
	ERBB2	15	PAK3	7		
	FGF1	11	PIAS1	5		
**hsa-miR-132-3p**	ARF6	3	RAB5B	2	RAF1	5
	CCNA2	5	RASA1	3	SIRT1	3
	CDKN1A	5	RB1	5	SPRED1	3
	CRK	5	FGF2	5	SPRY1	5
	CCNB1	3	GNB2L1	3	USP8	2
	EGFR	3	GNB1	2	WNT3A	3
	NBN	2	MAPK1	5		
